# Mechanism of Hsp70 activation: How J-domain proteins push for ATP hydrolysis

**DOI:** 10.1371/journal.pcbi.1014094

**Published:** 2026-03-19

**Authors:** Michał Olewniczak, Marcin Pitek, Jacek Czub, Jaroslaw Marszalek, Łukasz Nierzwicki, Bartlomiej Tomiczek

**Affiliations:** 1 Department of Physical Chemistry, Gdansk University of Technology, Gdansk, Poland; 2 Intercollegiate Faculty of Biotechnology, University of Gdansk, Gdansk, Poland; 3 BioTechMed Center, Gdańsk University of Technology, Gdańsk, Poland; University of Maryland School of Pharmacy, UNITED STATES OF AMERICA

## Abstract

Hsp70 chaperones are crucial for maintaining protein homeostasis by regulating the stability and conformational states of client polypeptides through ATP dependent cycles of binding and release. These cycles are driven by conformational transitions in Hsp70 upon ATP binding and hydrolysis. The ATPase activity of Hsp70 is controlled by J-domain protein (JDP) cochaperones, which allosterically stimulate ATP hydrolysis through interactions between their J-domains (JDs) and Hsp70. The JD binds at the interface between the nucleotide binding domain (NBD) and substrate binding domain (SBD) of ATP bound Hsp70. While it is well-established that JD interaction involves the conserved histidine-proline-aspartic acid (HPD) motif and residues in helices II and III, the mechanism by which JD-induced allosteric signals propagate to the distal nucleotide-binding pocket - and the conformational changes that facilitate ATP hydrolysis - remains unclear. Here, we addressed these questions using all-atom free energy simulations and dynamic network analysis, starting from crystal structures of ATP-bound DnaK (Hsp70) alone and in complex with the JD of DnaJ (JDP). We show that JD binding rearranges the NBD nucleotide-binding pocket into a hydrolysis competent state, characterized by the formation of a contact between the hydroxyl group of the universally conserved threonine 199 (T199) and the γ-phosphate of ATP. Network analysis revealed that the allosteric signal driving this rearrangement propagates along *β*-strands 13 and 14 towards T199. Moreover, we provide a mechanistic understanding for this signal transmission, demonstrating that steric repulsion between JD helix III and the SBD, alongside *β*-strand 14 disruption, facilitate T199-ATP contact formation. Overall, our study provides mechanistic insights into allosteric signal transmission within Hsp70, bridging the gap between JD binding and ATPase stimulation.

## Author summary

Hsp70 chaperones, together with J‑domain protein (JDP) co-chaperones, play a central role in maintaining protein homeostasis across diverse cell types and subcellular compartments. By coupling ATP binding and hydrolysis to conformational change, Hsp70s assist in protein folding, trafficking, degradation, and protein-protein interactions. Despite extensive study, the molecular basis by which JDPs stimulate the ATPase activity of Hsp70 has remained a challenging question. Here, using the crystal structure of Hsp70 bound to the J-domain of its JDP partner, we performed molecular dynamic simulations and complementary thermodynamic analyses to elucidate how JDP binding reshapes Hsp70 to promote ATP hydrolysis. Our results show that JDP binding generates a “steric push” that initiates an allosteric signal cascade, propagated through coordinated global and local conformational rearrangements. These structural changes reposition a conserved threonine residue closer to the ATP molecule, thereby facilitating catalysis. Our findings establish a structural and energetic framework linking JDP binding to the stimulation of Hsp70 ATP hydrolysis, providing mechanistic insight into a central step in chaperone function.

## Introduction

**Fig 1:**
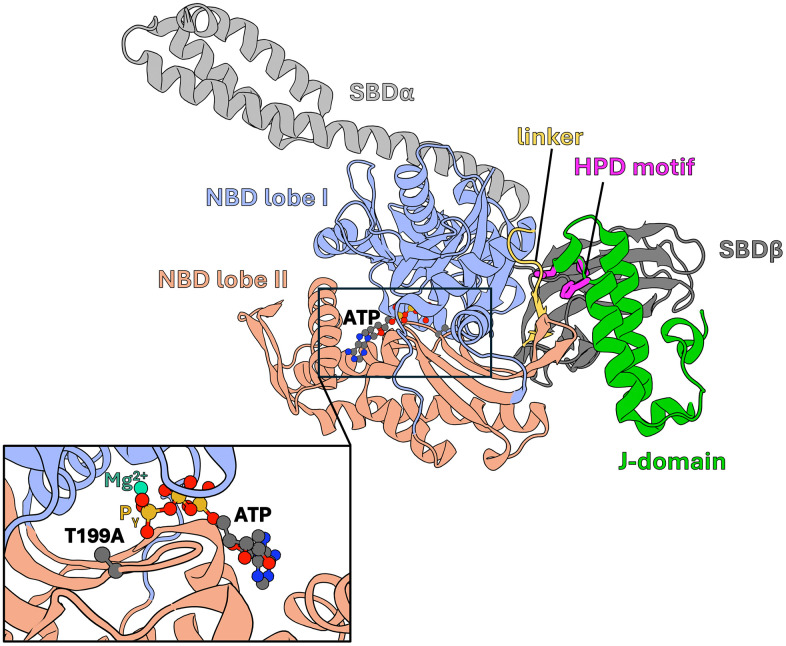
Structure of DnaK – DnaJ J-domain complex (PDB: 5NRO) in cartoon representation (NBD lobe I – light blue, NBD lobe II – light red, interdomain linker – yellow, SBD*β*– dark grey, SBDα – light grey, J-domain – green). J-domain’s HPD motif in licoricey representation – magenta. ATP in CPK representation coloured according to the elements (carbon – dark grey, nitrogen – blue, oxygen – red, phosphorus – orange). The insert shows the position of T199A substitution in relation to the ATP and Mg^2+^ (teal).

Cells are frequently exposed to environmental stressors, such as temperature, oxidative stress, toxic substances or radiation, which can disrupt protein homeostasis and cause protein aggregation, leading to cell death. Hsp70 chaperones, together with J-domain protein (JDP) cochaperones, play a critical role in controlling the conformational states of other proteins. Hsp70 machinery promotes protein folding into functional native conformations through ATP-dependent cycles of client protein binding and release [1–6]. The client binding cycle of Hsp70s is tightly regulated by interactions with the J-domain (JD) of their JDP cochaperone partners, which stimulate ATP hydrolysis [7]. In the ATP-bound state, the nucleotide binding domain (NBD) of Hsp70, which binds and hydrolyses ATP, is tightly docked to the substrate binding domain (SBD) and the interdomain linker (Fig 1). JDP binds client proteins and recruits Hsp70 in the ATP-bound state [6,8].

Binding of both the client and JD to Hsp70 stimulates ATP hydrolysis by inducing an allosteric transition from the restraining (R) state, in which hydrolysis is suppressed, to the stimulating (S) state, in which hydrolysis proceeds rapidly. Whereas the R-to-S transition has been extensively characterized for client binding, the conformational changes induced by JD binding remain incompletely defined and lack a detailed mechanistic explanation [9].

Following ATP hydrolysis to ADP, the SBD undocks from the NBD and the JD dissociates. Undocking is associated with the closure of α-helical lid subdomain of the SBD (SBDα) on the

*β*-sandwich subdomain (SBD*β*), which stabilizes the Hsp70-client interaction [10]. The cycle completion by ADP-to-ATP exchange is facilitated by another class of cochaperones known as Nucleotide Exchange Factors (NEFs). ATP rebinding subsequently triggers conformational changes that restore Hsp70 to the ATP-bound state [11–13].

Despite extensive studies, the mechanisms by which the JD allosterically communicates with residues in the nucleotide-binding pocket to stimulate ATP hydrolysis remain poorly understood [14–16]. Threonine 199 (T199), located within this pocket, likely interacts directly with the γ-phosphate of ATP, as suggested by its auto-phosphorylation and the marked reduction in ATPase activity observed upon its substitution with alanine (T199A) [15–18]. In many biomolecular motors with ATPase domains related to the NBD, threonine residues are critical for ATP hydrolysis by stabilizing the metaphosphate intermediate via side-chain mediated interactions [19,20]. While the crystal structure of the DnaK T199A variant in complex with the JD (PDB: 5NRO) reveals a potential network of contacts connecting the JD to the nucleotide-binding pocket, crystallographic data provide limited insight into T199 the mechanistic role in ATP hydrolysis, and the associated conformational dynamics remain unresolved [15].

It is well-established that upon JD binding, the universally conserved histidine-proline-aspartic acid (HPD) motif, located on the loop connecting helices II and III, plays a critical role in ATPase activation [21,22]. Evidence indicates that HPD-dependent displacement of the conserved R167 residue in the NBD, triggers Hsp70 conformational transitions that drive ATP hydrolysis [15,18]. Furthermore, studies of individual and combined HPD residue substitutions revealed its essential role in the Hsp70 client-binding cycle both in vitro and in vivo [22,23]. However, in some Hsp70/JDP systems, even a triple HPD/AAA variant is insufficient to fully abolish JD-dependent ATPase activation [24], indicating that the HPD motif alone cannot account for allosteric signalling. Therefore, residues within helices II and III, which are known to contribute to Hsp70 partner recognition, may also play important roles in this allosteric communication.

To elucidate the molecular mechanism by which the JD transmits the allosteric signal for ATP hydrolysis, we reverted the T199A substitution in the crystal structures of DnaK (Hsp70) both alone (Hsp70) and in complex with the JD of DnaJ (Hsp70-JD) and conducted all-atom free-energy simulations alongside dynamic network analysis. These analyses led us to propose a novel “steric push” mechanism, in which JD binding triggers a conformational transition of Hsp70, facilitating the displacement of T199’s hydroxyl group towards the γ-phosphate of ATP and thereby promoting hydrolysis.

## Results and discussion

### JD induces conformational changes in Hsp70

**Fig 2:**
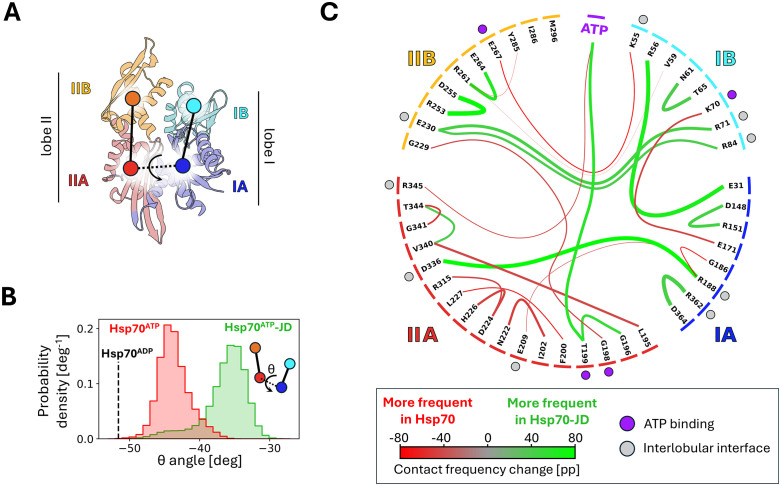
(A) Structure of the Hsp70’s NBD (DnaK) coloured by the subdomains (PDB: 5NRO). The subdomain IA of NBD is depicted in blue, IB– in cyan, IIA– in red and IIB– in orange. Dots indicate subdomain centres of mass, used to define torsion angle describing subdomain dynamics. (B) Probability distribution of the torsion angle between the NBD subdomains during MD simulations of the Hsp70 (red) and Hsp70-JD complex (green). Black dashed line indicates an angle value adopted by the Hsp70 in the ADP state (PDB: 2KHO). (C) Chord diagram showing changes in NBD residues contact frequency associated with Hsp70-JD complex. Line widths are proportional to the contact frequency in Hsp70-JD complex and are coloured based on the contact frequency difference with respect to Hsp70. Only contacts displaying frequency difference of 40 percentage points or greater are shown. Dots positioned next to the NBD residues indicate ATP-binding residues (violet) or residues positioned at the interlobular interface (grey) in the Hsp70-JD complex. Coloured bars along the circle’s rim indicate NBD subdomain.

To characterize the conformational changes in Hsp70 upon JD binding, we conducted unbiased all-atom molecular dynamics (MD) simulations based on crystal structures of DnaK T199A (PDB: 4JNE) in isolation and in complex with the JD of DnaJ (PDB: 5NRO), in which the T199 residue was restored. To identify the essential motions of Hsp70, we performed principal component analysis (PCA) on structural ensembles derived from the MD simulations. The first principal component, accounting for 28% of the conformational variability of the NBD (S2 Fig), was sufficient to distinguish between the NBD states of Hsp70 alone and in complex with JD (S1 Fig). Notably, the values of the first principal component were highly correlated with the torsion angle between the NBD lobes (R^2^ = 0.95), indicating that JD binding decreases the inter-lobe angle by approximately 7° relative to Hsp70 alone, resulting in a more compact NBD conformation (Fig 2A, 2B).

To further characterize the conformational changes in Hsp70 resulting from JD binding, we compared the frequency of inter-residue interactions - including hydrogen bonds, ionic interactions, *π*-stacking, *π*-cation interactions and hydrophobic contacts - in Hsp70 alone and in the Hsp70-JD complex, with a focus on the NBD (Fig 2C). Notably, the most significant differences in contact frequency were observed in: (i) the JD binding site, (ii) the ATP-binding site, and (iii) lobe IIA (see also S3-S4 Figs). Strikingly, in the Hsp70-JD complex we observed an interaction between the hydroxyl group of T199 and the γ-phosphate of ATP that is absent in Hsp70 alone (S5 Fig). Analogous interaction has been observed in many ATPases, where it contributes to stabilizing of the metaphosphate intermediate during ATP hydrolysis [19,20].

Additionally, our simulations of the Hsp70-JD complex revealed further local rearrangements within the nucleotide-binding pocket. Notably, displacement of the Y145 sidechain from the SBD*β* interface towards the ATPase catalytic site occurs more frequently in the presence of the JD, suggesting that this rearrangement may affect the transition towards the T199-close state (S6 Fig). Our observations are consistent with recent studies describing the client-binding-induced transition of DnaK T199A between the restraining (R) and stimulating (S) states [9,25]. This transition involves rotation of the NBD lobes and displacement of residue Y145 towards the γ-phosphate of ATP [9] (S20 Fig). Our results also capture the displacement of the G-loop (residues 196–199) observed in the crystal structure of the DnaK T199A S-state: following JD binding, G198 no longer interacts with G229, whereas G196 forms an interaction with T199 (Figs 2C, S4).

In parallel, several residues previously implicated in allosteric communication between the NBD and SBD - including K70, D148, R151, E171 - exhibit substantial changes in their interaction patterns in the Hsp70-JD complex compared with Hsp70 alone (S4 Fig) [26–28]. Specifically, the loss of contact between E171 and K70 in Hsp70-JD complex may allow E171 to coordinate the attacking water molecule during ATP hydrolysis [15]. Finally, our results suggest that the compact NBD conformation induced by JD binding is stabilized by the formation of inter-lobe salt bridge involving residues R71, R84, E230 and switching the R188 interaction partner from E209 to D336.

Taken together, our MD simulations of the ATP bound Hsp70-JD complex with restored T199 reveal the allosteric effects of JD binding, which involves conformational transitions within the NBD. These transitions include rotation of the NBD lobes and reorganization of the NBD contact network, notably the displacement of T199 towards the *γ*-phosphate of ATP.

### JD facilitates the positioning of T199 next to the γ-phosphate of ATP

**Fig 3:**
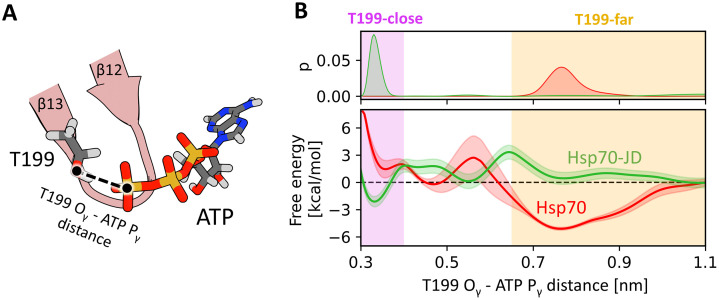
(A) Schematic representation of the reaction coordinate used in the umbrella sampling simulations. The atoms shown in licoricey representation are coloured according to the elements – carbon in dark grey, oxygen in red, hydrogen in light grey, phosphorus in orange, nitrogen in blue. (B) Probability distributions (top) and free energy profiles (bottom) for the distance between hydroxyl oxygen of T199 and the phosphorus of ATP gamma phosphate in the absence (red) and presence (green) of the JD. The purple shade indicates the T199-close state (distance < 0.4 nm), whereas orange shade indicates the T199-far state (distance > 0.65 nm).

Our unbiased MD simulations revealed that JD binding favours displacement of T199 towards the γ-phosphate of ATP. To thermodynamically characterize this conformational transition, we determined free-energy profiles for T199 movement towards the γ-phosphate of ATP in both Hsp70 alone and in complex with JD, using enhanced sampling methods (Fig 3). We employed a well-established umbrella sampling approach, performing simulations until convergence was achieved.

Comparison of the resulting energy profiles indicates that the positioning of T199 strongly depends on the presence of JD. For Hsp70 alone, the free energy profile shows a well-defined minimum corresponding to a state in which the hydroxyl group of T199 is displaced from the γ-phosphate of ATP by ∼0.8 nm (T199-far state). Formation of the T199 - γ-phosphate contact in this state incurs an energy cost of ~6 kcal/mol, indicating that, in the absence of the JD, the T199 sidechain is unlikely to participate in stabilization of the ATP-to-ADP transition state.

In contrast, in the Hsp70-JD complex, the free energy profile exhibits a pronounced global minimum at ∼0.35 nm (T199-close state). In this state, formation of the T199 - γ-phosphate contact is energetically favourable, associated with a free-energy gain of ~3 kcal/mol. These results indicate that the T199-close state is predominantly adopted in the presence of JD, as reflected in the probability distribution along the T199 - γ-phosphate distance (Fig 3B). By contrast, in the absence of JD, the T199-close state is rarely accessible, being 6 kcal/mol less favourable than the T199-far state.

To further assess the role of Y145 in the transition to the T199-close state, we performed additional umbrella sampling simulations for the Hsp70 Y145A variant, both in isolation and in complex with JD (S21-S22 Figs). In the presence of JD, the free energy profiles showed only minor changes in the depth or position of the free-energy minima compared to the wild-type Hsp70-JD complex, indicating a limited role of Y145 in the transition to the T199-close state. Interestingly, the Y145A substitution reduced the free energy barrier for this transition (at ~0.6 nm of the T199- γ-phosphate of ATP distance) by ~2–3 kcal/mol. In contrast, the free-energy profile for isolated Hsp70 Y145A was flattened for the T199-far and intermediate distances. These findings are consistent with previous biochemical data showing that DnaK Y145A^10^, exhibits higher ATPase activity than the wild-type, both in the presence and absence of DnaJ, highlighting the functional relevance of the T199-close state. Collectively, our results support a model in which the JD binding induces rearrangements in the nucleotide binding pocket, promoting T199 interaction with the ATP γ-phosphate and thereby explaining ATPase stimulation.

### Allosteric signalling between JD and nucleotide-binding pocket of Hsp70

To elucidate how JD interaction at the surface of Hsp70 induces conformational changes within the nucleotide-binding pocket on the opposite side of the NBD [15,18], we first investigated the contribution of HPD motif residues to allosteric signalling. We focused on H33 and D35, which are known to form direct contacts with L391 and R167 of the NBD, respectively [15]. MD simulations of the Hsp70-JD H33A complex revealed an equilibrium conformation that closely resembles the wild-type complex. Consistently, the free energy profile for JD H33A binding to Hsp70 was nearly indistinguishable from that of the wild-type JD (S14 Fig;left), indicating that the H33A substitution does not impair JD-Hsp70 interaction. However the assessment of H33A effect on the transition to the T199-close state showed that the T199-far state is 3 kcal/mol more stable than the T199-close state (S14 Fig;right). These results demonstrate that although the H33A substitution preserves JD binding, it shifts conformational equilibrium towards the T199-far state, thereby preventing Hsp70 from adopting the stimulating (S) state.

Next, we examined the effects of the D35A substitution. MD simulations of the Hsp70-JD D35A complex showed that only helix II of JD remains in direct contact with the NBD, whereas helix III and the interhelical loop are displaced further from the Hsp70 binding site compared to the wild-type complex (S12, S13 Figs). Consistent with this observation, the free energy profile along the JD-Hsp70 distance (S14 Fig) indicates that D35A binding is ∼2 kcal/mol less favourable and leads to a relaxation of the complex, as evidenced by ∼0.2 nm shift in the free energy minimum. We further investigated the impact of the D35A substitution on the stability of the SBD*β*-NBD interface. In previous studies, JD binding was shown to partially displace SBD*β* from NBD [18]. However, umbrella sampling simulations of the Hsp70-JD D35A complex revealed that this displacement is abolished (S18 Fig). These findings underscore the critical role of the salt bridge between D35 of HPD motif and R167 of the NBD [18] in driving the conformational transition required for ATP hydrolysis. Thus, although the D35A and H33A substitutions differently affect JD binding, both prevent Hsp70 from undergoing the conformational transition to the stimulating (S) state, albeit through distinct mechanisms.

Because H33 and D35 within the HPD motif directly interact with L391 of the interdomain linker and R167 of the NBD, respectively, we hypothesised that local α-helix disruption by P34 enables H33 and D35 to adopt orientations that favour these interactions [29]. To test this hypothesis, we performed MD simulations of the isolated JD and its P34A variant. As expected, the P34A substitution extends the α-helical structure of helix II into the HPD motif. Therefore, a dihedral angle describing the relative orientation of H33 and D35 displays a broad distribution in the P34A variant, whereas in the wild-type JD this angle is narrowly distributed around values close to 0**°**, indicating that both residues face the same direction (S11 Fig). Together, these results demonstrate that P34 plays a key role in constraining orientation of H33 and D35, thereby enabling their productive interactions with L391 and R167.

Finally, to assess the combined effects of the HPD substitutions, we performed umbrella sampling simulations of Hsp70 in complex with JD HPD/AAA variant. The resulting free energy profile along the Hsp70-JD distance lacks an energetically favourable minimum (S15 Fig), indicating that the triple HPD/AAA substitution abolishes formation of a stable Hsp70-JD complex. Consistent with the effects observed for individual HPD substitutions, this triple JD mutant prevents formation of the stable Hsp70-JD complex required for Hsp70 to transition into the stimulating (S) state.

**Fig 4:**
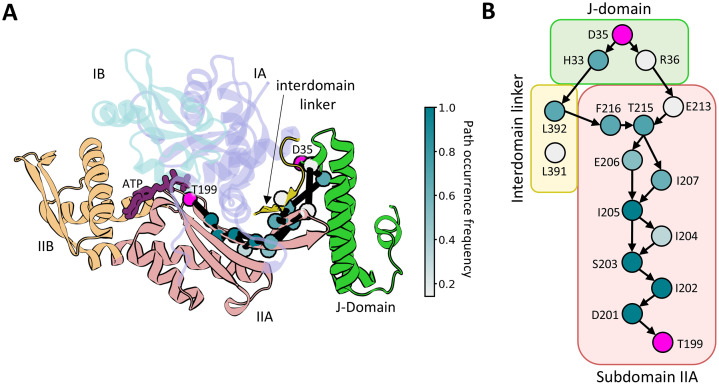
Allosteric signalling between JD and Hsp70. (A) Set of 5 optimal pathways between the D35 of the JD HPD motif and the T199 of Hsp70 (magenta spheres) calculated for 5 Hsp70-JD MD trajectories and projected onto the Hsp70-JD complex. C_α_ atoms of residues present in the set of optimal and suboptimal pathways shown as spheres and coloured according to the path occurrence frequency (for more details see S7 Fig). (B) Graph depicting connectivity of pathway forming residues. Arrows represent connections present in the set of 5 optimal pathways, residue colouring as in (A).

To elucidate the allosteric signal transmission routes connecting the HPD motif to T199, we performed additional analyses using graph theory, an approach that has proven effective in revealing allosteric communication in biomolecules [30,31]. We constructed a dynamic network model, in which the protein is represented as a network of C*α* atoms (residues) interconnected by edges whose lengths are determined by the strength of correlated residue motions (connections). Analysis of our multi-µs MD trajectories revealed that all identified allosteric pathways follow a similar pattern: the signal originating at D35 is relayed via H33, within the HPD motif, propagates through the interdomain linker and NBD *β*-strands 13 and 14 (residues F200–T215), and ultimately converges on T199 (Figs 4, S7). Notably, 7 of the 14 residues involved in these pathways have previously been implicated in allosteric communication by mutational and structural studies [22,27,32–34].

Interestingly, none of the identified pathways directly involve the well-established interaction between D35 of the HPD motif and R167 of the NBD, which has been shown to be critical for stimulation of ATP hydrolysis [15]. Given that the otherwise non-functional DnaK R167A mutant is suppressed by the compensatory DnaJ D35N substitution both in vivo and in vitro [23,35], we next examined how these mutations affect the allosteric pathways described above. In the mutant complex, all identified pathways follow trajectories similar to those observed in the wild-type system (S24 Fig). Consistently, free-energy calculations comparing the T199-far and T199-close states for the Hsp70 R167A-JD D35N variant reveal a flattened free energy landscape, rendering the T199-close state more accessible than in Hsp70 alone (S23 Fig). Together, these results indicate that suppression of the R167A mutation by the D35N substitution does not reroute the allosteric communication network.

To further evaluate the proximity of the JD to T199 of the NBD in correlation space, we employed community network analysis [30,36], which simplifies the dynamic network by clustering strongly connected nodes into communities. The resulting community structure broadly reflects the division of the NBD into its constituent subdomains (S8–S9 Figs). T199 was assigned to the zeroth community, which is directly connected to the first community containing the JD. This organization provides a structural rationale for the observed routing of allosteric pathways between D35 of the JD and T199, as minimizing community crossings likely reduces path length in correlation space.

In addition, three leucine residues in the conserved VLLL motif of the interdomain linker were assigned to the same community as the JD, highlighting the tight coupling between these regions. We next quantified the strength of inter-community connections using the betweenness centrality (BC) metric (see Methods) and identified key interactions involved in allosteric signal transmission based on connections with the highest BC values. Both IA and IIA subdomains of the NBD establish strong connections with the JD. Specifically, JD residues D35, K26, Y25, H33 and F47 interact with NBD residues R167, E217, L391 and L392. Notably, D35 and H33 of the HPD motif form direct contacts with R167 of the NBD and L391 of the interdomain linker, respectively (S10 Fig). Interactions of H33 with L391 or L392 facilitate transmission of the allosteric signal towards the catalytic site (Figs 4, S10) [15].

### JD-induced allosteric signalling is associated with destabilization of Hsp70’s *β*-strand 14 induced by steric repulsion

Analysis of our unbiased MD simulations revealed multiple events in which the T199-close state coincided with disruption of the interaction between *β*-strand 13 (containing T199) and *β*-strand 14 (residues 198–206 and 219–227; S19A Fig), accompanied by destabilization of the *β*-strand 14 itself. Probability distributions indicate that these disruption events are more frequent in the Hsp70-JD complex than in Hsp70 alone (S19B Fig). Notably, conformational changes in the *β*13-*β*14 region have previously been identified as structural signatures distinguishing the restraining (R) and stimulating (S) states of Hsp70 [9].

To examine whether these conformational changes are sufficient to drive the transition towards the T199-close state, we performed free energy simulations along the T199–ATP distance while enforcing two distinct conformations of the *β*13-*β*14 region. First, we enforced a disrupted conformation in Hsp70 alone (see Methods for more details). The resulting free-energy profile (S17 Fig; left) closely resembles that of native Hsp70, indicating that disruption of the *β*-strands is not sufficient to promote the T199-close state. Second, we enforced an undisrupted conformation in the Hsp70-JD complex. The corresponding profile shows a clear preference for the T199-far state (S17 Fig; right), demonstrating that the maintenance of an intact *β*-strands conformation prevents the transition to the T199-close state even in the presence of the JD. Taken together, these results indicate that disruption of the of the *β*13-*β*14 region is necessary, but not sufficient, for the transition to the T199-close state.

**Fig 5:**
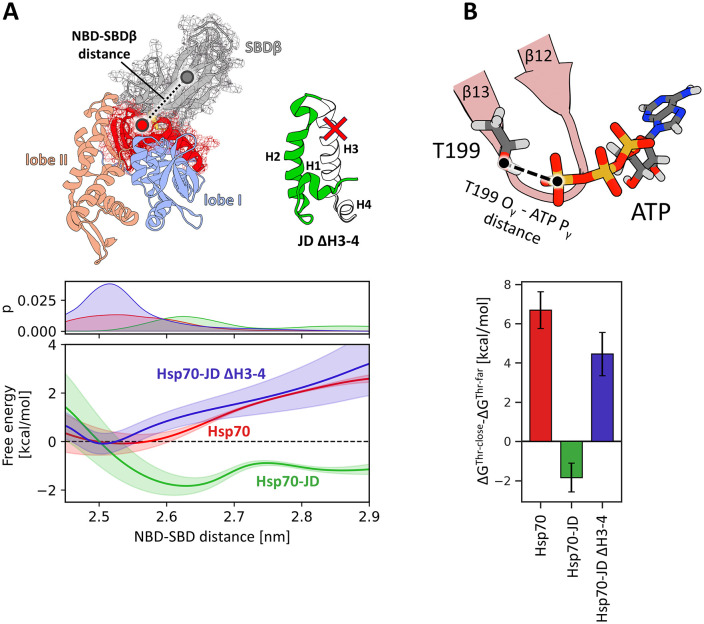
Role of the helix III of JD. (A) Free energy profiles for the dissociation of the NBD and SBD*β*. The profile for the Hsp70 without JD is depicted in red, for the complex of Hsp70 and wild-type JD– in green, whereas for the complex of Hsp70 with the JD lacking helix III and IV– in blue. The definition of the distance between centres of mass of the SBD*β* and the part of NBD interfacing SBD*β* is shown above. (B) Differences of the free energy associated with the transition from the T199-far to T199-close state for the same systems as in 4A (for the free energy profiles see S16 Fig).

Knowing that disruption of the *β*13-*β*14 region alone is insufficient to reach the T199-close state and that tight JD binding is required for allosteric signal transmission, we hypothesised that in the Hsp70-JD complex, steric repulsion from the JD exerts a mechanical push that drives both displacement of the SBD*β* from the NBD and disruption of the *β*-strands. To test this, we constructed an Hsp70 complex with a JD variant lacking helix III and IV (residues 41–65), reasoning that the JD ∆H3-4 variant would bind Hsp70 but be unable to exert the mechanical push. Indeed, free energy profiles along the SBD*β-*NBD and T199-ATP distances for the Hsp70-JD ∆H3-4 variant are similar to those of Hsp70 alone, indicating that the truncated JD prevents SBD*β* displacement from the NBD (Fig 5A) and maintains the T199-far state (Fig 5B). These findings support our hypothesis that steric repulsion exerted by the intact JD is necessary for allosteric signal transmission.

## Conclusions

**Fig 6:**
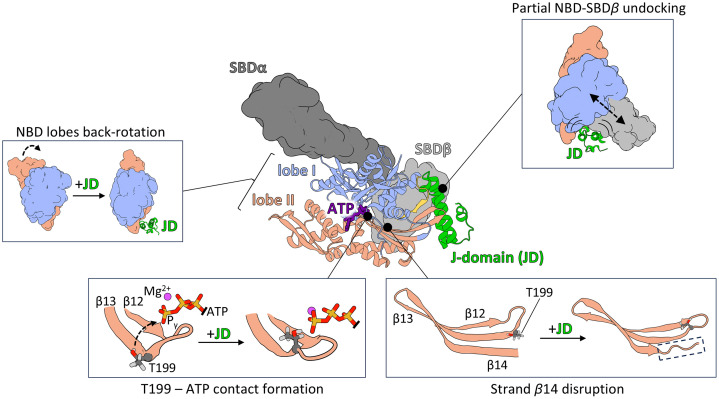
Schematic representation of the mode of action of the JD in the stimulation of ATPase activity. By tight binding to Hsp70, JD exerts both local and global conformational changes within Hsp70. Globally JD promotes partial NBD-SBD*β* undocking, which diminish the SBD*β* inhibitory effect on NBD lobes rotation reducing the torsion angle between them. Locally the JD binding initiates propagation of allosteric signal through *β*-sheet in subdomain IIA, which results in contact formation between T199 and gamma phosphate of ATP and disruption of *β*-sheet’s strand 14. Altogether observed rearrangements prime the Hsp70’s NBD for the ATP hydrolysis. The SBD*β* is depicted in light grey, SBDα – in dark grey, NBD lobes I and II – in light blue and salmon respectively and the JD– in green. The atoms shown in licoricey representation are coloured according to the elements – carbon in dark grey, oxygen in red, hydrogen in light grey, phosphorus in orange and magnesium in magenta.

We propose that JD binding to Hsp70 generates a steric push that induces both global and local conformational changes, resulting in stimulation of ATPase activity (Fig 6). This push promotes partial undocking of the SBD*β* from the NBD surface, allowing back-rotation of the NBD lobes and, consequently, rearrangement of the contact network within the NBD. These rearrangements involve residues in the nucleotide-binding pocket that are critical for the ATP hydrolysis. Tight binding of the intact JD is required for this push, as substitutions in the HPD motif, or truncation of the JD (deletion of helices III and IV) abolish these global conformational changes.

At the local level, JD-binding initiates propagation of an allosteric signal from the D35 and H33 of the HPD motif through conserved residues of the inter-domain linker and *β*-strand 14 into *β*-strand 13, which contains the conserved T199 residue essential for ATP hydrolysis. This allosteric signal also causes partial disruption of *β*-strand 14, which is necessary for transition from the T199-far state to the T199-close state. In the T199-close state, the side chain hydroxyl of T199 forms a hydrogen bond with the γ-phosphate of ATP, thereby contributing to stabilization of the transition state during hydrolysis.

Although the critical role of T199 in ATP hydrolysis is supported by numerous biochemical experiments involving substitutions of this residue [17,26], structural insight into the mechanism of T199 involvement has so far been lacking. This gap arises because all structural analyses of the ATP-bound Hsp70s have been performed using T199A substitution variants [7,9,15,37].

However, conformational rearrangements of the *β*-sheet, formed by *β*-strands 13 and 14 were also revealed by the crystal structure of DnaK in complex with a client peptide [9]. This finding implies that both client and JD binding can induce conformational changes within NBD. Crucially, within the Hsp70–JD complex, these changes favour the formation of the T199-close state and, consequently, promote ATP hydrolysis.

It is well-established that JD and client polypeptides synergistically stimulate Hsp70 ATPase activity [8,10,38]. We hypothesise that the allosteric pathways triggered by both JD and client binding converge on T199 and promote its transition towards the T199-close state. Further studies involving Hsp70 simultaneously engaged with both JD and client will be required to elucidate how these allosteric signals are integrated and why ATPase activity is stimulated many-fold only in the presence of both ligands.

## Methods

### Simulation protocol

All simulations were performed using Gromacs 5.0.4 [39] with the Plumed 2.1 plugin [40]. CHARMM36m force field [41] was used for proteins, ions and Mg-ATP, and the TIP3P model [42] was used for water. In each of the simulation boxes, the numbers of Na^+^ and Cl^−^ ions were adjusted to 0.15 M. Temperature was kept at 310 K with the v-rescale algorithm [43] using a coupling constant of 0.1 ps. Pressure was kept at 1 bar using the Parrinello-Rahman algorithm [44] with a coupling time of 5 ps. Periodic boundary conditions were applied and the Particle Mesh Ewald summation [45] was used to calculate long-range electrostatic interactions with a cut-off radius of 1 nm and a Fourier grid spacing of 0.12 nm. Van der Waals interactions were calculated with Lennard-Jones potential with a cut-off radius of 1 nm. All bonds involving hydrogen were constrained using the LINCS algorithm [46]. Leap-frog Verlet algorithm [47] was used to integrate equations of motion with a time step of 2 fs.

The energy of the systems was minimized in two stages using steepest descent algorithm: at first, all heavy atoms of the protein were kept fixed; in the second step only the position of the protein backbone was restrained, allowing protein side chains to relax [48,49]. After that, the systems were relaxed with short MD simulation (10 ns) with constrained protein backbone.

### Conventional MD simulations

The Hsp70 system was prepared using 4JNE PDB structure [37] placed in a ∼13.5 nm x 13.5 nm x 13.5 nm box solvated with ∼55000 water molecules. The system was simulated in two copies and for each copy the 2.5 *µ*s-long trajectory was obtained. The Hsp70-JD system was prepared using 5NRO PDB structure [15] placed in a ∼13 nm x 13 nm x 13 nm box solvated with ∼52000 water molecules. The system was simulated in five copies and for each copy the 1.8 *µ*s-long trajectory was obtained. In each system, the crystal water molecules coordinated to Mg^2+^ ion in Mg-ATP were kept. As the terminal region of SBD*α* is dispensable for the transmission of the allosteric signal [9,50] to reduce the size of the system and thus, to allow for the better convergence of the free energy profiles, the C-terminal region of the lid (residues 534–609) was removed. The final structures from these simulations were used as the initial configurations for the subsequent free energy calculations.

The wild-type and P34A JD systems (residues 3–65) were prepared using JD from the 5NRO PDB structure [15]. Each system was placed in the ∼6 nm x 6 nm x 6 nm box, solvated with ∼7000 water molecules and simulated for 1 *µ*s.

### Trajectories analysis

Principal Component Analysis was performed for the backbone of the Hsp70’s NBD (residues 2–384) with gmx covar and gmx anaeig tools [39]. The eigenvectors for JD-induced NBD conformational changes were assigned based on the merged NBD trajectories from Hsp70 and Hsp70-JD systems. The merged trajectory was then projected onto the first eigenvector.

Torsion angle of NBD subdomains was defined as the angle between centre of mass of the backbone atoms of lobes IB (residues 40–115), IA (residues 1–39, 116–188, 361–384), IIA (residues 189–228, 307–360) and IIB (residues 229–306) [51].

Contact analysis was performed using GetContacts tool [52] for the NBD and interdomain linker of Hsp70 (residues 2–394) using merged trajectories of Hsp70 and Hsp70-JD complex. The interactions were classified as differentiating if the difference in contact frequency between Hsp70 and Hsp70-JD systems was greater than 40 percentage points. For defining inter-residue contacts following types of interactions were considered: hydrogen bonds, ionic interactions, *π*-stacking, *π*-cation interactions and hydrophobic interactions (defined as Van der Waals interactions between purely hydrophobic residues). Default GetContacts criteria were used for all interaction types with exception of the criteria for the hydrogen bond detection were the α_DH···A_ angle value greater than or equal to 150 ° was required. Calculated contact frequencies were then visualized as chord diagrams using the Flareplot tool [52]. NBD residues were classified as interacting with ATP if they remained in contact for at least 50% of the trajectory. Residues were considered to be at the NBD interlobular interface if they remained in contact with residues from the opposite NBD lobe for at least 50% of the trajectory.

Dynamic network analysis was performed with dynetan 1.0.1 python package [31]. Merged trajectories of Hsp70-JD system were used to infer common network topology and perform community and betweenness centrality analyses. For tracing allosteric pathways between JD D35 and DnaK T199 weights of connections in the network were calculated using individual trajectories as separate analysis windows, while keeping previously obtained network topology. Nodes of the network were defined as the Cα atoms positions of the NBD and interdomain linker (residues 2–394) or JD (residues 3–65). Additional nodes were assigned for the magnesium ion associated with ATP molecule in the catalytic site of Hsp70, as well as for the ATP itself (two nodes, matching the positions of N1 atom of purine ring and terminal phosphorus atom). Nodes (residues) were interconnected by edge (connection) if any heavy atoms from two nodes remained within 0.45 nm from each other for at least 75% of the simulation length. In the case of ATP, the heavy atoms of the purine ring were assigned to the N1 atom node, whereas heavy atoms of phosphate groups and ribose were assigned to the γ-phosphate node. For each connection, the weight of the connection was calculated as the generalized correlation (GC) coefficient, which captures both linear and nonlinear correlations between residues.

Clustering of the densely interconnected nodes into communities was performed using the Louvain method [53], taking into the account the weight of the connection. Communities that made up for less than 1% of all the nodes were discarded in further analysis. The strength of the connection between the communities was quantified using the betweenness centrality measure, defined as the fraction of the shortest pathways, between all pairs of nodes, passing through a selected node, showing which nodes play a bridging role in the network.

For each of the simulated replicas, the allosteric pathways between D35 from JD conserved HPD motif (“source”) and T199 of NBD (“sink”) were calculated using Floyd-Warshall algorithm [54]. For given connection between atoms *i* and *j*, the distance parameter *d*_*ij*_, defined as *d*_*ij*_= − log(*GC*_*ij*_), was calculated and the optimal path was assigned in such a way that the sum of distances along the pathway from the “source” to “sink” was minimized. Additionally, twenty sub-optimal pathways were determined. Based on the calculated pathway set, the residue pathway occurrence frequency was calculated. To confirm that the general conclusions from the pathway’s analysis are not dependent on the values of the parameters used to construct the network, we performed additional sensitivity analysis (S42-S44 Figs). The connections in new networks were assigned by taking into the account different thresholds, both for the distance cut-off between heavy atoms (4.2-4.8 Å), as well as for the contact persistence (65–85%). Then, pathway similarity was assessed using Jaccard index (overlap between sets of pathways, measured as residue overlap and connection overlap).

The antiparallel beta sheet content (*s*) of residues 198–206 and 219–227 of Hsp70 was calculated using ANTIBETARMSD method [55] from Plumed plugin with default parameter values and strands cutoff equal to 1. In the wild-type and P34A JD systems the torsion angle between H33 and D35 residues was defined as the angle between H33 N*∊*, H33 C*β*, D35 C*α* and D35 C*γ*. The probability distributions for distance between Helix III of JD (residues 41–58) and SBD*β* (residues 395–505) and the distance between HPD motif and R167 were calculated using gmx distance and gmx mindist tools [39], respectively.

All molecular images were created using VMD and Blender [56,57].

### Free energy calculations

All of the free energy profiles were computed with the replica-exchange umbrella sampling (REUS) method [58] and determined using the weighted histogram analysis method [59]. Uncertainties of the free energy profiles were estimated using bootstrap error analysis taking into account the autocorrelation in the analysed time series. (for the convergence of the calculated profiles see S25-S41 Figs).

### Conformational change in the catalytic site of NBD

The free energy profiles associated with the T199 conformational change in the catalytic site of the NBD were calculated along the reaction coordinate defined as the distance between the T199 hydroxyl oxygen and the terminal phosphorus atom of ATP. The initial configurations for REUS simulations were obtained with 80 ns-long steered-MD simulation, during which the distance between T199 and ATP was gradually increased using a moving one-sided potential with a force constant of 2000 kJ· mol^−1^· nm^−2^. Ten equally spaced US windows were used, spanning the range of 0.3 to 1.2 nm of the reaction coordinate. The spring constant of the harmonic biasing potential was set to 1500 kJ· mol^−1^· nm^−2^ and each of the REUS windows was simulated for 1 *µ*s. The same procedure was applied for the substitution variants.

During the investigation of the beta-sheet disruption influence on the T199-far to T199-close transition additional simulations were performed. In the Hsp70-JD system, the beta-sheet was restrained by setting the parameter *s* to 2.73, whereas in the Hsp70 system the beta-sheet was disrupted by keeping the *s* parameter at the value of 1.8. In both cases the one-sided harmonic potential was used, with the spring constant of 4000 kJ· mol^−1^· nm^−2^.

In the case of the JD ∆H3-4 system, during the free energy calculations the conformation of the remaining JD (residues 3–40) was restrained with the additional harmonic biasing potential (with the spring constant set to 5000 kJ· mol^−1^· nm^−2^), which kept the 0.0 nm RMSD value with respect to the wild-type helix II conformation.

### JD positioning on Hsp70

The free energy profiles associated with the JD binding on Hsp70 were calculated along the reaction coordinate defined as the distance between the centre of mass of backbone atoms of the JD (residues 25–31 and 48–54) and the centre of mass of C*α* atoms of Hsp70 binding site (residues 208–216 and 415–422). The initial configurations for REUS simulations were obtained with 100 ns-long steered-MD simulation, during which the distance between Hsp70 and JD was gradually increased using a moving one-sided harmonic potential with a force constant of 1500 kJ· mol^−1^· nm^−2^. Six equally spaced US windows were used, spanning the range of 0.8 to 1.3 nm of the reaction coordinate. The spring constant of the harmonic biasing potential was set to 3500 kJ· mol^−1^· nm^−2^ and each of the REUS windows was simulated for 800 ns.

### SBD and NBD dissociation

In the studies of SBD and NBD dissociation the reaction coordinate was defined as the distance between the centre of mass of C*α* atoms of the SBD*β* domain (residues 395–505) and the centre of mass of C*α* atoms of the NBD fragments, which are in direct contact with SBD*β* (residues 146–226). To make the system significantly smaller and thus, to achieve better convergence of the free energy profiles, the lid sub-domain, SBD*α*, was truncated by removing residues 506–602 of Hsp70.

The initial configurations for REUS simulations were obtained with 500 ns-long steered MD simulations, during which the distance between NBD and SBD*β* was gradually increased using a moving one-sided potential with a spring constant of 2500 kJ· mol^−1^· nm^−2^, from the initial value of 2.58 nm up to 3.58 nm. Five equally spaced US windows were used, spanning the range of 2.5 to 2.9 nm of the reaction coordinate. The spring constant of the harmonic biasing potential was set to 3500 kJ· mol^−1^· nm^−2^ and each of the REUS windows was simulated for 1 *µ*s.

## Supporting information

S1 Fig Distribution of the Hsp70 and Hsp70-JD trajectories projected onto the first eigenvector obtained by the Principal Component Analysis. The visual representation of the NBD along the first eigenvector is depicted on the right.
(TIF)

S2 Fig Results of the Principal Component Analysis for the merged Hsp70 and Hsp70-JD trajectories. (A) Percentage of the total variance explained by each of the first five eigenvectors. (B) Correlation between the values of the first component and the torsion angle of the NBD subdomains.
(TIF)

S3 Fig Residues of Hsp70 which display the most prominent differences in the contact frequency within NBD and inter-domain linker in the presence or absence of the JD. Colour scheme: purple– ATP molecule, blue– lobe IA of NBD, cyan– lobe IB of NBD, red– lobe IIA of NBD, orange– lobe IIB of NBD, yellow– interdomain linker. Only NBD of DnaK showed for clarity.
(TIF)

S4 Fig Chord diagram showing changes in NBD residues contact frequency associated with Hsp70 alone. Line widths are proportional to the contact frequency in Hsp70 alone and are coloured based on the contact frequency difference with respect to Hsp70-JD complex. Only contacts displaying frequency difference of 40 percentage points or greater are shown. Dots positioned next to NBD residues indicate ATP-binding residues (violet) or residues positioned at the interlobular interface (grey) in the Hsp70 alone. Coloured bars along the circle’s rim indicate NBD subdomain containing a given residue.
(TIF)

S5 Fig Probability distribution of the distance between oxygen from the hydroxyl group of T199 and the γ-phosphate of ATP during conventional MD simulations of the Hsp70 (red) and Hsp70-JD complex (blue). (TIF)

S6 Fig Probability distribution of the distance between hydrogen from the hydroxyl group of Y145 and the oxygen from the hydroxyl group of T199 of Hsp70 during conventional MD simulations of the Hsp70 (red) and Hsp70-JD complex (blue). (TIF)

S7 Fig Allosteric paths connecting JD’s D35 to DnaK’s T199 (magenta, in vdw representation) determined for each of the simulated replica of Hsp70-JD system. The optimal path is shown in blue, whereas suboptimal ones are shown in red. ATP is shown in dark violet using licoricey representation.
(TIF)

S8 Fig Structural (top) and graph (bottom) representation of the community analysis for the Hsp70-JD complex. The width of the connections between communities in the bottom panel corresponds to the betweenness centrality measure between them (for exact values see S1 Table) and the circle size to the number of residues belonging to a given community. Communities are numbered from 0 to 10. The ATP atoms belong to communities 0 and 2.
(TIF)

S9 Fig Composition of the communities obtained during the community network analysis of the Hsp70-JD complex. In the case of NBD, the lobes of NBD, which correspond to each residue range were indicated on the right. The -1 community represents nodes which were not assigned to any community.
(TIF)

S10 Fig Pair of residues forming edges having the highest betweenness centrality on the JD-DnaK interface. Only edges with betweenness value greater than 0.01 are shown. Colour scheme of the proteins interface on the right corresponds to the communities composition in S9 Fig. Edge forming residues on the JD are in green, residues on the DnaK are in magenta.
(TIF)

S11 Fig Probability distribution of the dihedral angle between H33 and D35 in the wild-type JD (blue) and the P34A variant (violet) of JD. (TIF)

S12 Fig Dissociation of the D35A mutant of JD from the specific binding site. (A) Probability distribution of the distance between SBDβ and helix III of D35A variant (brown) and the wild-type (blue) JD (B) Position of the D35A variant of the JD bound to Hsp70 (brown) with respect to the wild-type bound JD (transparent blue).
(TIF)

S13 Fig Probability distribution of the minimum distance between R167 of Hsp70 and the HPD motif of JD for wild-type (blue) and D35A mutant (brown). (TIF)

S14 Fig (Left) The probability distributions (top) and free energy profiles (bottom) for the JD association to the Hsp70. The profile for the H33A mutant was shown in cyan, profile for the D35A mutant was shown in brown, whereas profile for the wild-type JD binding was shown in blue. (Right) The probability distributions (top) and free energy profiles (bottom) for the T199-far to T199-close conformational change for the complex of H33A mutant (cyan) or the wild-type (blue) JD bound to Hsp70.
(TIF)

S15 Fig The probability distributions (top) and free energy profiles (bottom) for the HPD/AAA mutated variant of JD association to the Hsp70. The profile for the H33A, P34A, D35A mutant was shown in orange, profile for the wild-type in blue.
(TIF)

S16 Fig The probability distributions (top) and free energy profiles (bottom) for the T199-far to T199-close conformational change for the ∆H3-4 JD (magenta). Profiles for the Hsp70 (blue) and Hsp70-JD (red) were added for comparison.
(TIF)

S17 Fig (Left) The probability distributions (top) and free energy profiles (bottom) for the T199-far to T199-close conformational change in the absence of the JD. The non-biased profile for the formed β-sheet is shown in red, whereas the profile for the bias-induced disrupted β-sheet is shown in green. (Right) The probability distributions (top) and free energy profiles (bottom) for the T199-far to T199-close conformational change in the presence of the JD. The non-biased profile for the disrupted β-sheet is shown in blue, whereas the profile for the bias-induced β-sheet kept is shown in magenta.
(TIF)

S18 Fig Probability distributions (top) and free energy profiles (bottom) for the dissociation of the NBD and SBDβ subdomains of Hsp70 caused by wild-type JD (blue) and D35A variant of JD (magenta). (TIF)

S19 Fig Disruption of the beta-sheet caused by the JD binding. (A) Structural representation of the movement of T199 towards the ATP enabled by beta-sheet disruption. The ATP and T199 are shown in licoricey representation, whereas beta-sheet is shown in cartoon representation. (B) Probability distribution of the antiparallel beta-sheet content of the 198–206 and 219–227 residues of Hsp70 in the Hsp70 alone (red) and the Hsp70-JD complex (blue).
(TIF)

S20 Fig Coordination of the γ-phosphate of ATP in the crystal structure of the substrate-induced stimulated-state of Hsp70 (PDB: 7KRU, left) and the T199-close state of Hsp70 visible in our simulations of the Hsp70 with JD (right). (TIF)

S21 Fig Free energy profiles of the T199-far to T199-close conformational change for the Y145A Hsp70 mutant (dark green) and wildtype (red). (TIF)

S22 Fig Free energy profiles of the T199-far to T199-close conformational change for the Y145A Hsp70-JD mutant (brown) and wildtype complex (blue). (TIF)

S23 Fig Probability distribution (top) and free energy profile (bottom) of the T199-far to T199-close conformational change for the Hsp70 (R167A) - JD (D35N) double mutant (green). Profiles for wildtype complex (blue) and Hsp70 alone (red) added for comparison.
(TIF)

S24 Fig Allosteric pathways determined for the Hsp70 (R167A) - JD (D35N) double mutant. The pathways between HPD motif of JD and the catalytic pocket of Hsp70 were calculated from the Umbrella Sampling trajectories corresponding to the T199-close state. The optimal pathway was indicated in blue, whereas suboptimal pathways were depicted in red.
(TIF)

S1 Table Normalized betweenness values connecting the communities in Hsp70-JD system. (XLSX)

S25 Fig Convergence of the Free energy profile along the T199 Oγ-ATP Pγ distance for the Hsp70 system as a function of the length of US trajectories used for analysis. (TIF)

S26 Fig Convergence of the Free energy profile along the T199 Oγ-ATP Pγ distance for the Hsp70-JD system as a function of the length of US trajectories used for analysis. (TIF)

S27 Fig Convergence of the Free energy profile along the T199 Oγ-ATP Pγ distance for the Hsp70-H33A JD system as a function of the length of US trajectories used for analysis. (TIF)

S28 Fig Convergence of the Free energy profile along the T199 Oγ-ATP Pγ distance for the Hsp70-JD ΔH3–4 system as a function of the length of US trajectories used for analysis. (TIF)

S29 Fig Convergence of the Free energy profile along the T199 Oγ-ATP Pγ distance for the Hsp70 system with disrupted β–sheet as a function of the length of US trajectories used for analysis. (TIF)

S30 Fig Convergence of the Free energy profile along the T199 Oγ-ATP Pγ distance for the Hsp70-JD system with formed β–sheet as a function of the length of US trajectories used for analysis. (TIF)

S31 Fig Convergence of the Free energy profile along the T199 Oγ-ATP Pγ distance for the R167A Hsp70-D35N JD system as a function of the length of US trajectories used for analysis. (TIF)

S32 Fig Convergence of the Free energy profile along the T199 Oγ-ATP Pγ distance for the Y145A Hsp70 system as a function of the length of US trajectories used for analysis. (TIF)

S33 Fig Convergence of the Free energy profile along the T199 Oγ-ATP Pγ distance for the Y145A Hsp70-JD system as a function of the length of US trajectories used for analysis. (TIF)

S34 Fig Convergence of the Free energy profile along the DnaK-JD distance for the Hsp70-JD system as a function of the length of US trajectories used for analysis. (TIF)

S35 Fig Convergence of the Free energy profile along the DnaK-JD distance for the Hsp70-H33A JD system as a function of the length of US trajectories used for analysis. (TIF)

S36 Fig Convergence of the Free energy profile along the DnaK-JD distance for the Hsp70-D35A JD system as a function of the length of US trajectories used for analysis. (TIF)

S37 Fig Convergence of the Free energy profile along the DnaK-JD distance for the Hsp70-H3A,P34A,D35A JD system as a function of the length of US trajectories used for analysis. (TIF)

S38 Fig Convergence of the Free energy profile along the NBD-SBD distance for the Hsp70 system as a function of the length of US trajectories used for analysis. (TIF)

S39 Fig Convergence of the Free energy profile along the NBD-SBD distance for the Hsp70-JD system as a function of the length of US trajectories used for analysis. (TIF)

S40 Fig Convergence of the Free energy profile along the NBD-SBD distance for the Hsp70-D35A JD system as a function of the length of US trajectories used for analysis. (TIF)

S41 Fig Convergence of the Free energy profile along the NBD-SBD distance for the Hsp70-JD ΔH3–4 system as a function of the length of US trajectories used for analysis. (TIF)

S42 Fig Sensitivity analysis of network topology and path routing between JD D35 and DnaK T199 to contact distance and persistence cutoffs. (A) Jaccard similarity (intersection of connections over union) between network topologies using default cutoff values (4.5 Å distance cutoff and 75% contact persistence cutoff) and the alternative cutoffs varied in 4.2-4.8 Å range for the distance and 65–85% for the contact persistence. (B) Similarity comparison of unique residue sets that form paths between JD D35 and DnaK T199. (C) Analogous analysis for sets of unique path-forming connections. (D) Occurrence frequency of the path forming residues, present in the original analysis, in the 8 analysed alternative cutoff combinations.
(TIF)

S43 Fig Sensitivity analysis of the pathway routing between D35 of JD and T199 of DnaK dependence on the chosen values of contact distance cutoff and contact persistence cutoff, performed only for a set of 5 optimal pathways. (A) Jaccard similarity for the sets of unique residues present in a set of optimal pathways derived from analyses with different cutoff values. (B) Frequency of residue occurrence in alternative analyses.
(TIF)

S44 Fig Routing of the optimal and 20 suboptimal pathways in 5 analysis windows for the networks defined by extreme cutoff values (4.2 Å distance, 85% persistence), (4.8 Å distance, 65% persistence). (TIF)

S45 Fig ToC. (TIFF)
