# Supplementary figures and images for: Mechanism of Hsp70 activation: How J-domain proteins push for ATP hydrolysis
Source: PLoS Comput Biol. 2026 Mar 19;22(3):e1014094. doi: 10.1371/journal.pcbi.1014094 (PMC13012618; doi:10.1371/journal.pcbi.1014094)

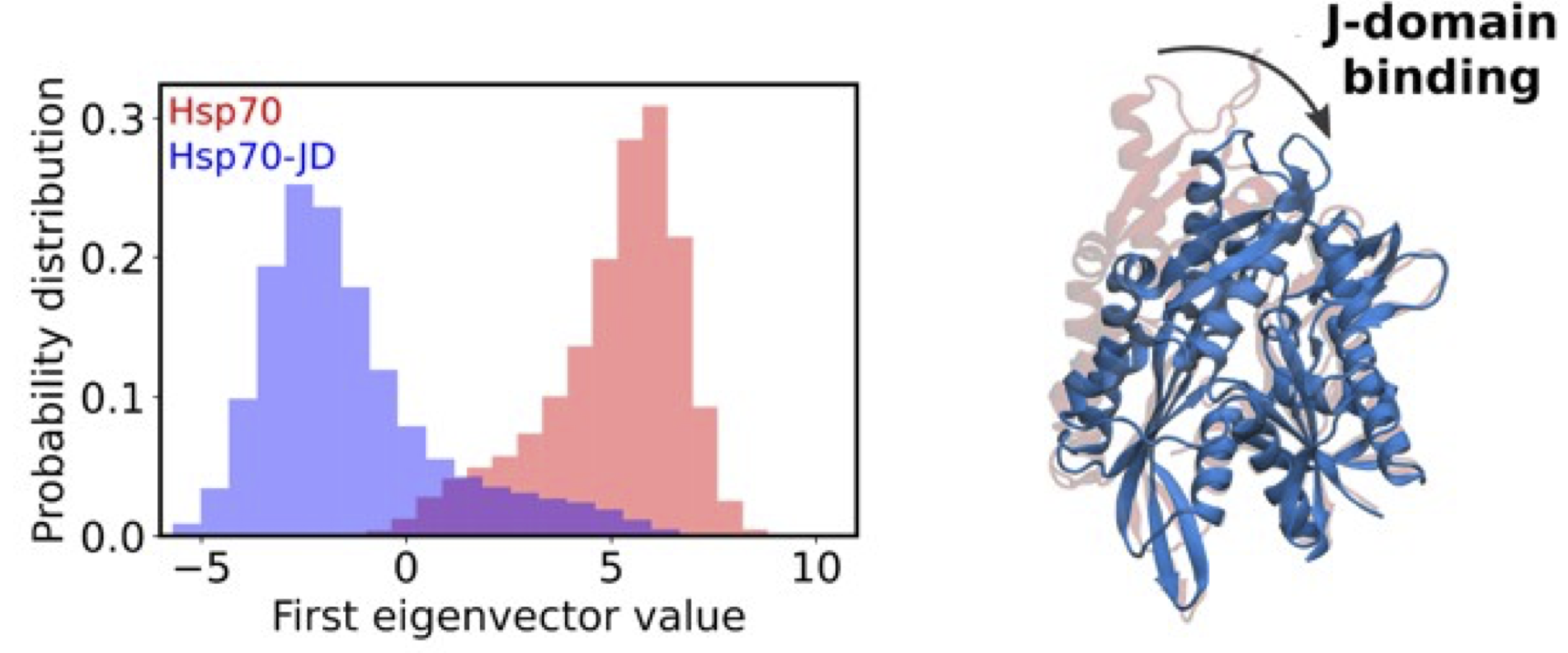

Supplement: S1 Fig — The visual representation of the NBD along the first eigenvector is depicted on the right. (TIF) [file pcbi.1014094.s001.tif]

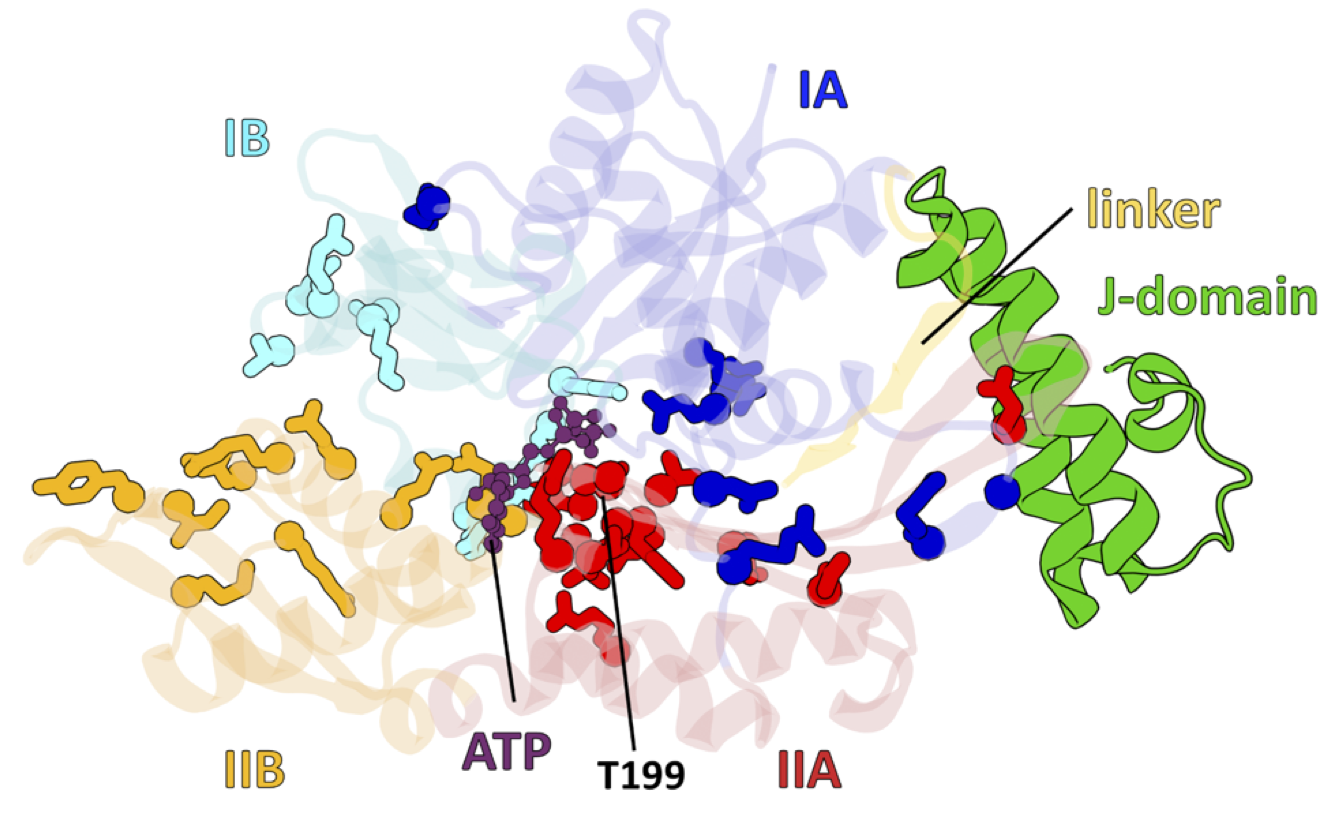

Supplement: S3 Fig — Colour scheme: purple– ATP molecule, blue– lobe IA of NBD, cyan– lobe IB of NBD, red– lobe IIA of NBD, orange– lobe IIB of NBD, yellow– interdomain linker. Only NBD of DnaK showed for clarity. (TIF) [file pcbi.1014094.s003.tif]

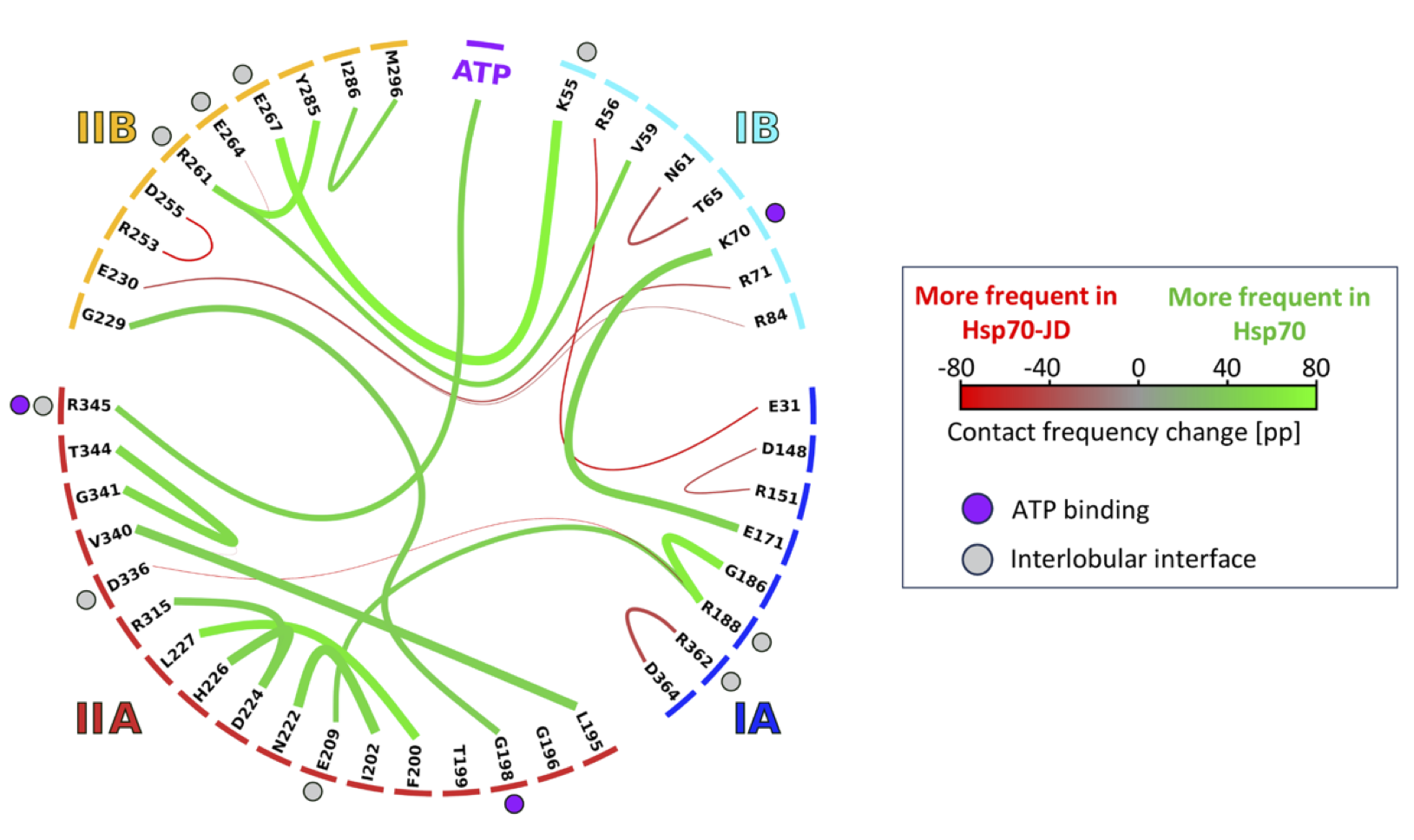

Supplement: S4 Fig — Line widths are proportional to the contact frequency in Hsp70 alone and are coloured based on the contact frequency difference with respect to Hsp70-JD complex. Only contacts displaying frequency difference of 40 percentage points or greater are shown. Dots positioned next to NBD residues indicate ATP-binding residues (violet) or residues positioned at the interlobular interface (grey) in the Hsp70 alone. Coloured bars along the circle’s rim indicate NBD subdomain containing a given residue. (TIF) [file pcbi.1014094.s004.tif]

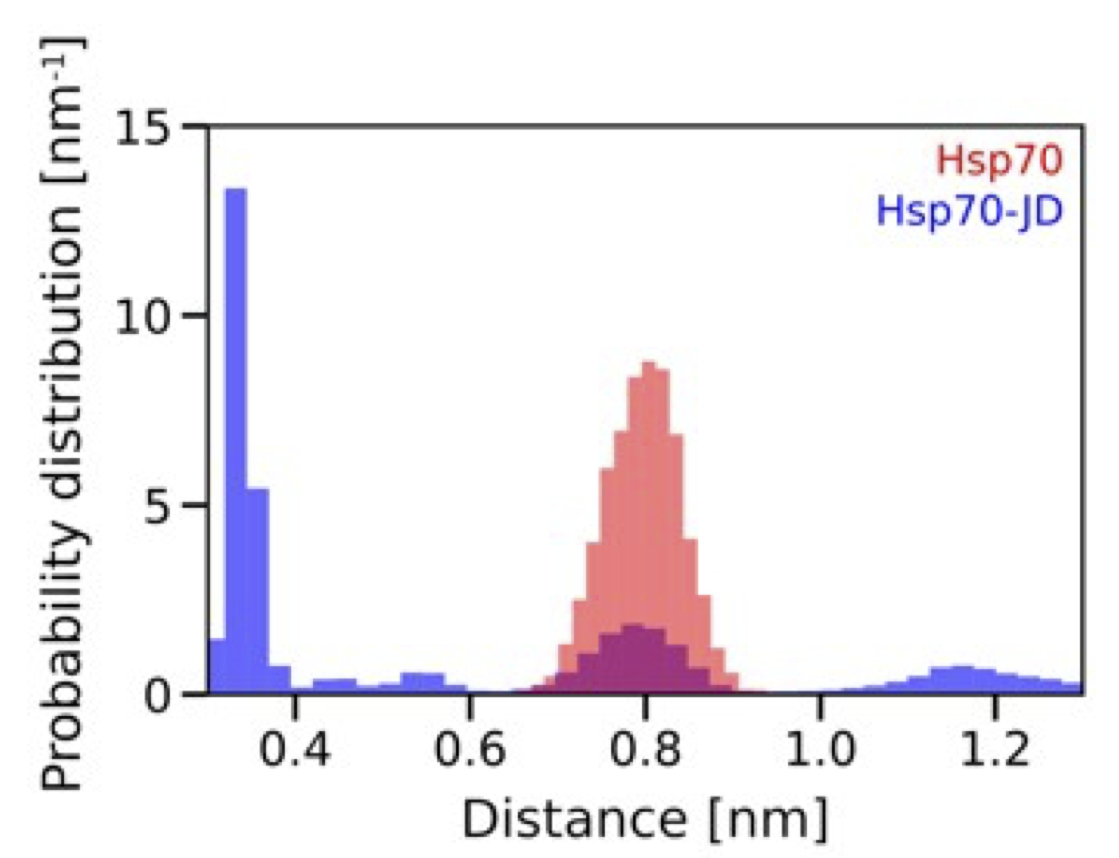

Supplement: S5 Fig — (TIF) [file pcbi.1014094.s005.tif]

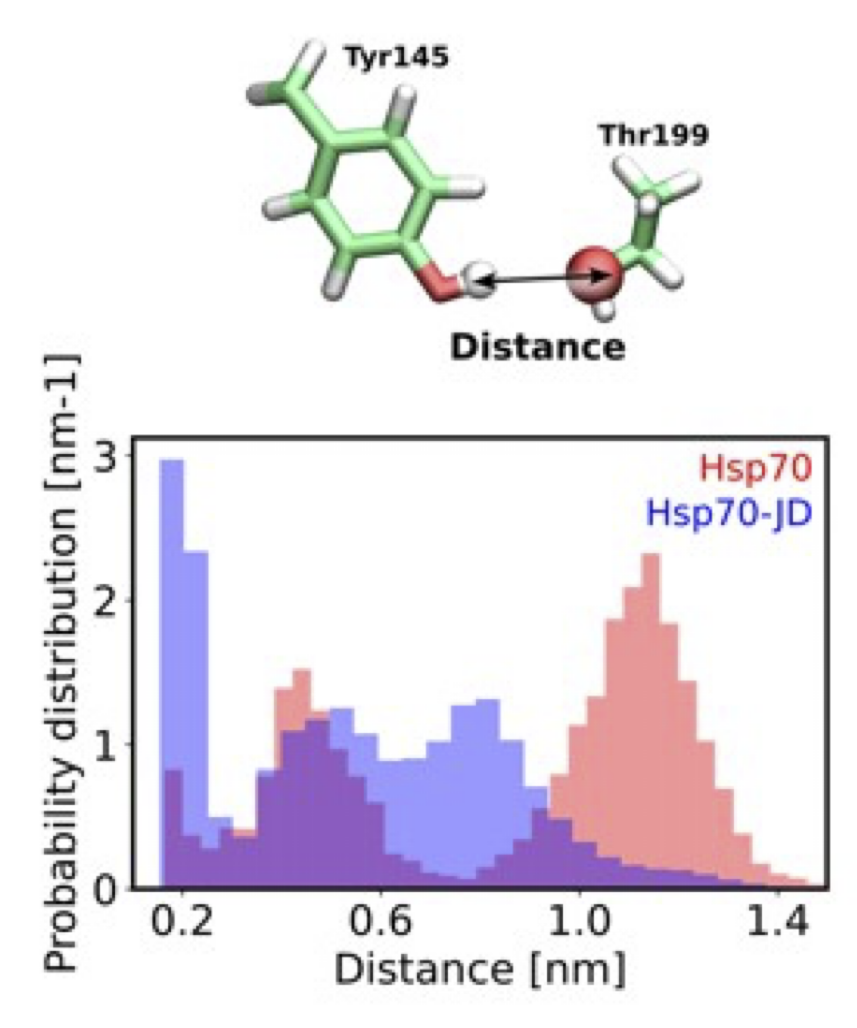

Supplement: S6 Fig — (TIF) [file pcbi.1014094.s006.tif]

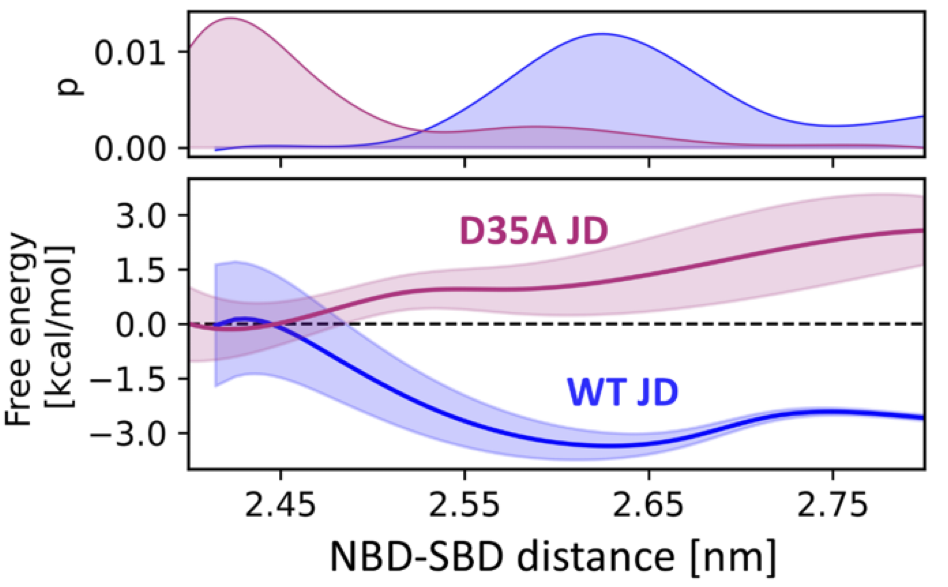

Supplement: S7 Fig — The optimal path is shown in blue, whereas suboptimal ones are shown in red. ATP is shown in dark violet using licoricey representation. (TIF) [file pcbi.1014094.s017.tif]

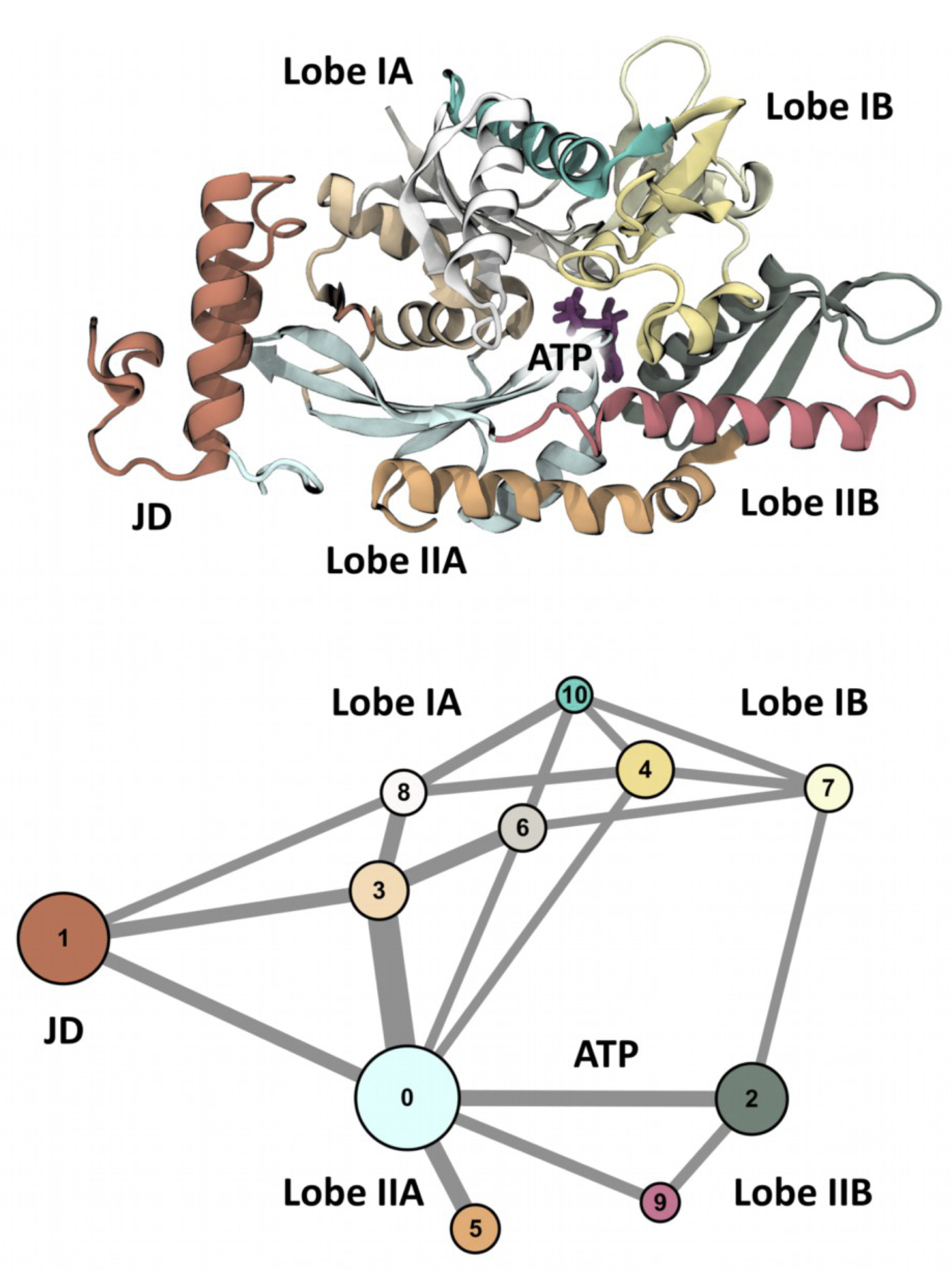

Supplement: S8 Fig — The width of the connections between communities in the bottom panel corresponds to the betweenness centrality measure between them (for exact values see S1 Table) and the circle size to the number of residues belonging to a given community. Communities are numbered from 0 to 10. The ATP atoms belong to communities 0 and 2. (TIF) [file pcbi.1014094.s007.tif]

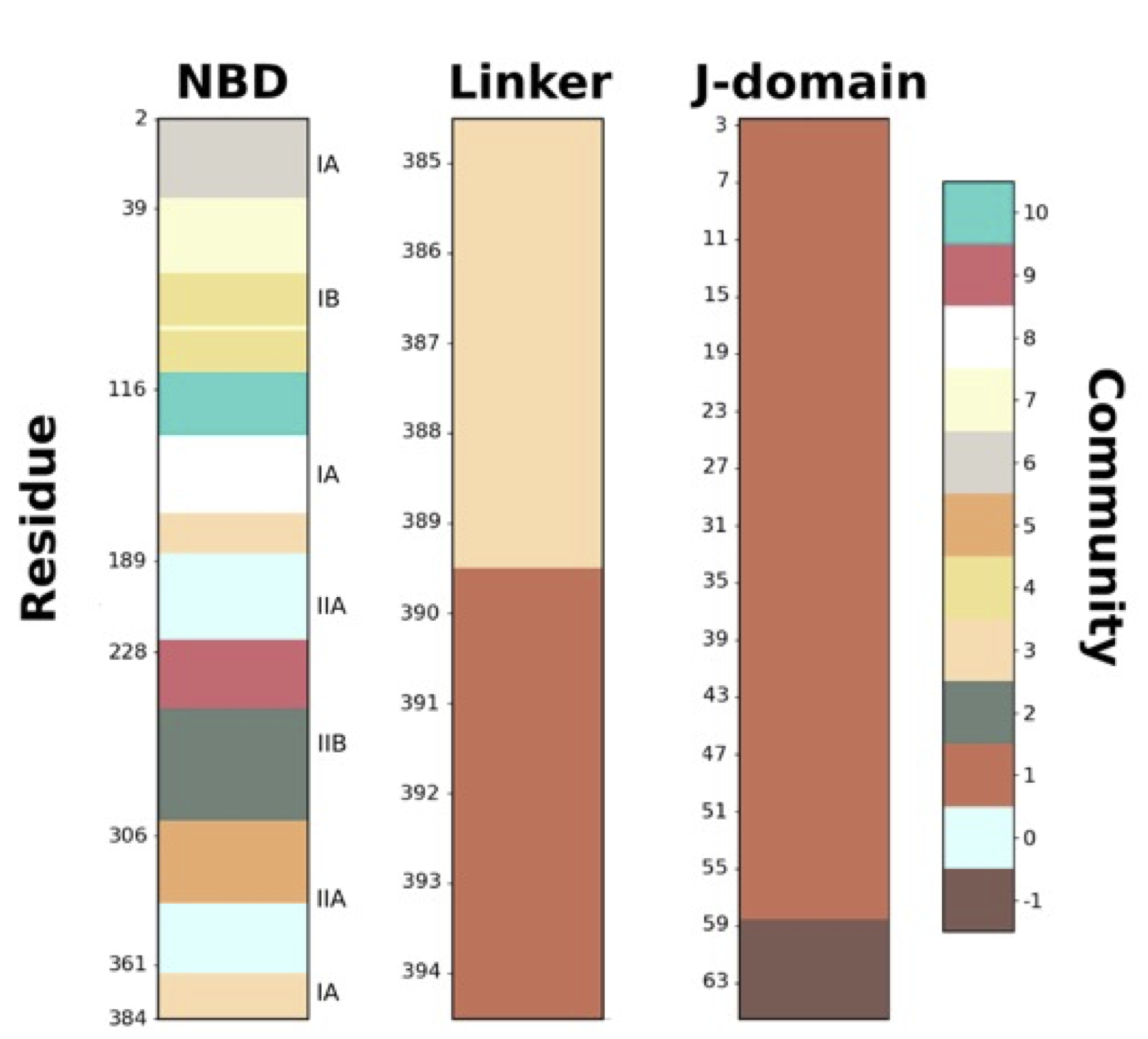

Supplement: S9 Fig — In the case of NBD, the lobes of NBD, which correspond to each residue range were indicated on the right. The -1 community represents nodes which were not assigned to any community. (TIF) [file pcbi.1014094.s008.tif]

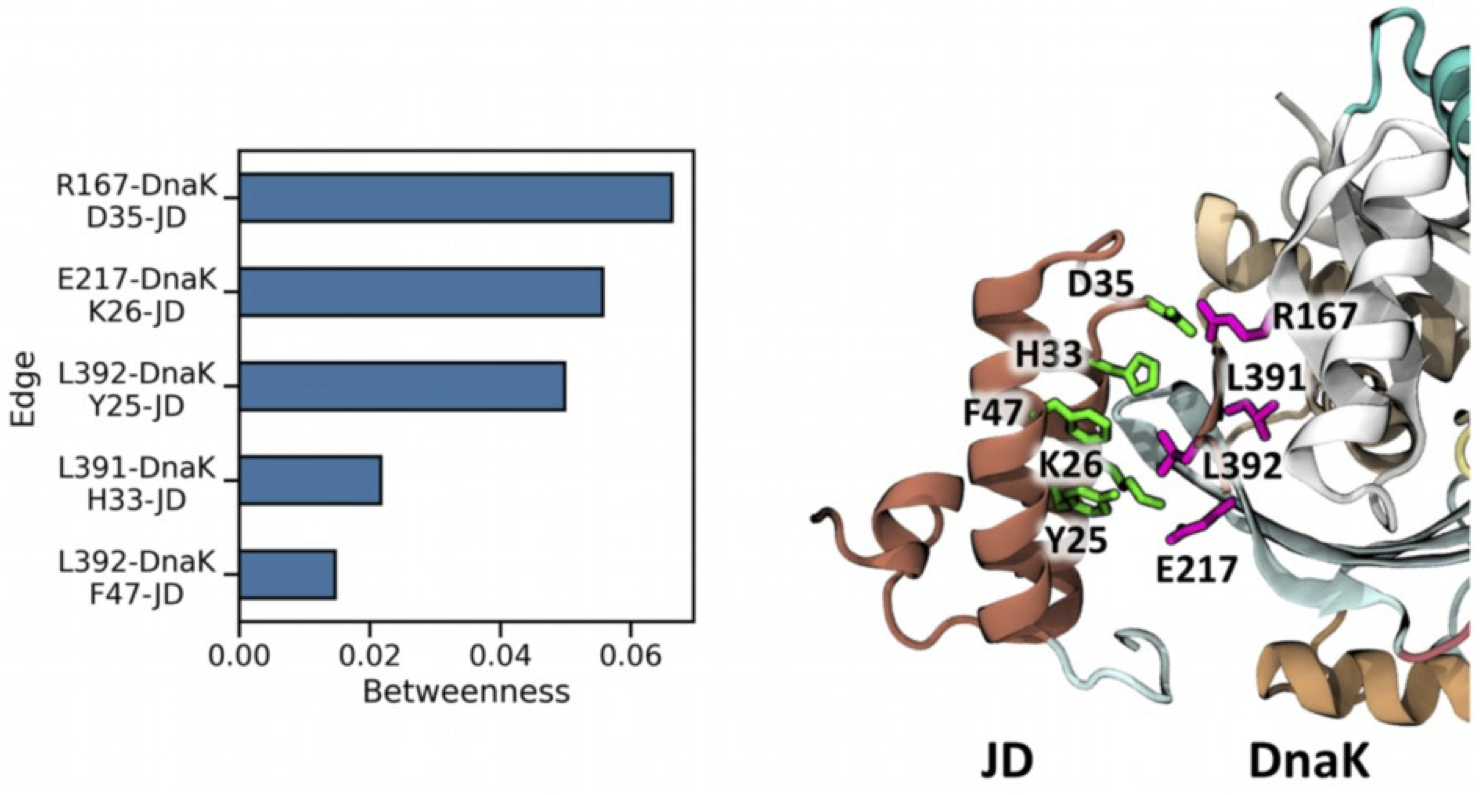

Supplement: S10 Fig — Only edges with betweenness value greater than 0.01 are shown. Colour scheme of the proteins interface on the right corresponds to the communities composition in S9 Fig. Edge forming residues on the JD are in green, residues on the DnaK are in magenta. (TIF) [file pcbi.1014094.s009.tif]

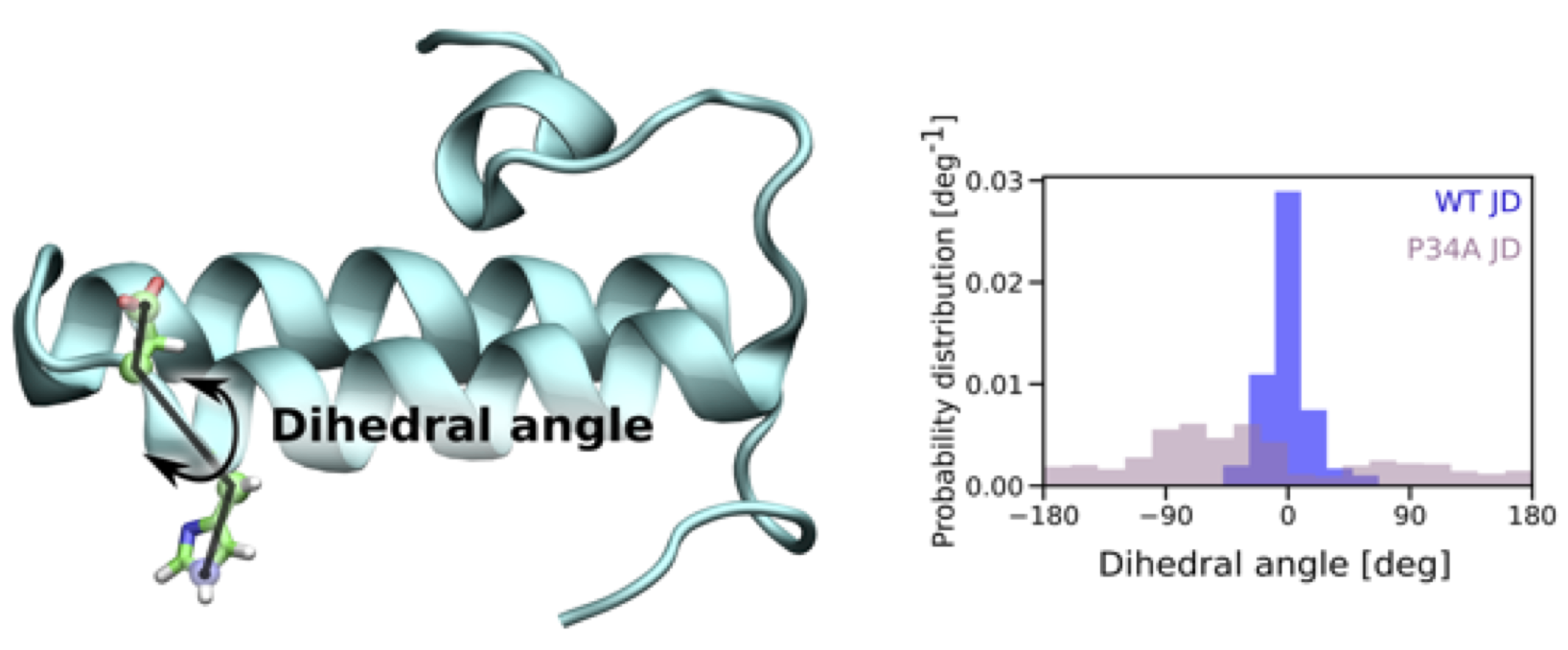

Supplement: S11 Fig — (TIF) [file pcbi.1014094.s010.tif]

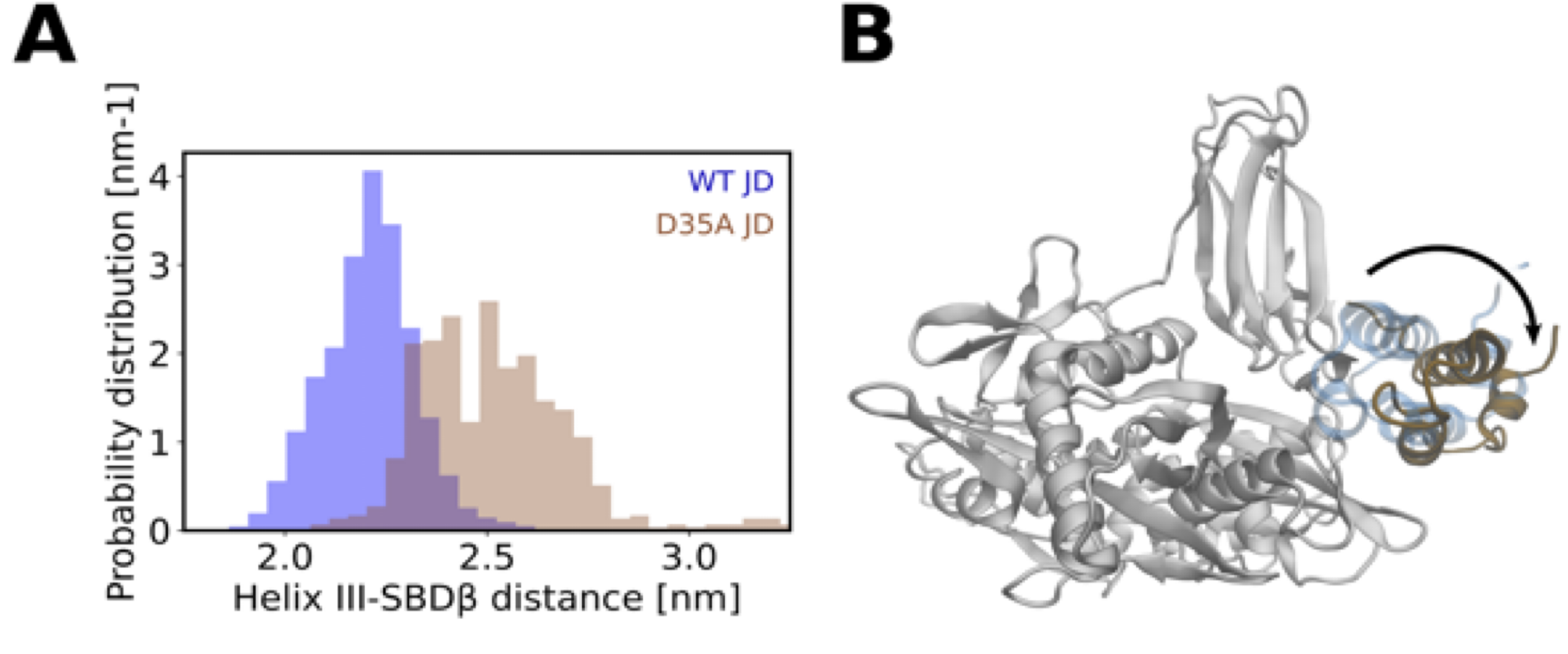

Supplement: S12 Fig — (A) Probability distribution of the distance between SBDβ and helix III of D35A variant (brown) and the wild-type (blue) JD (B) Position of the D35A variant of the JD bound to Hsp70 (brown) with respect to the wild-type bound JD (transparent blue). (TIF) [file pcbi.1014094.s011.tif]

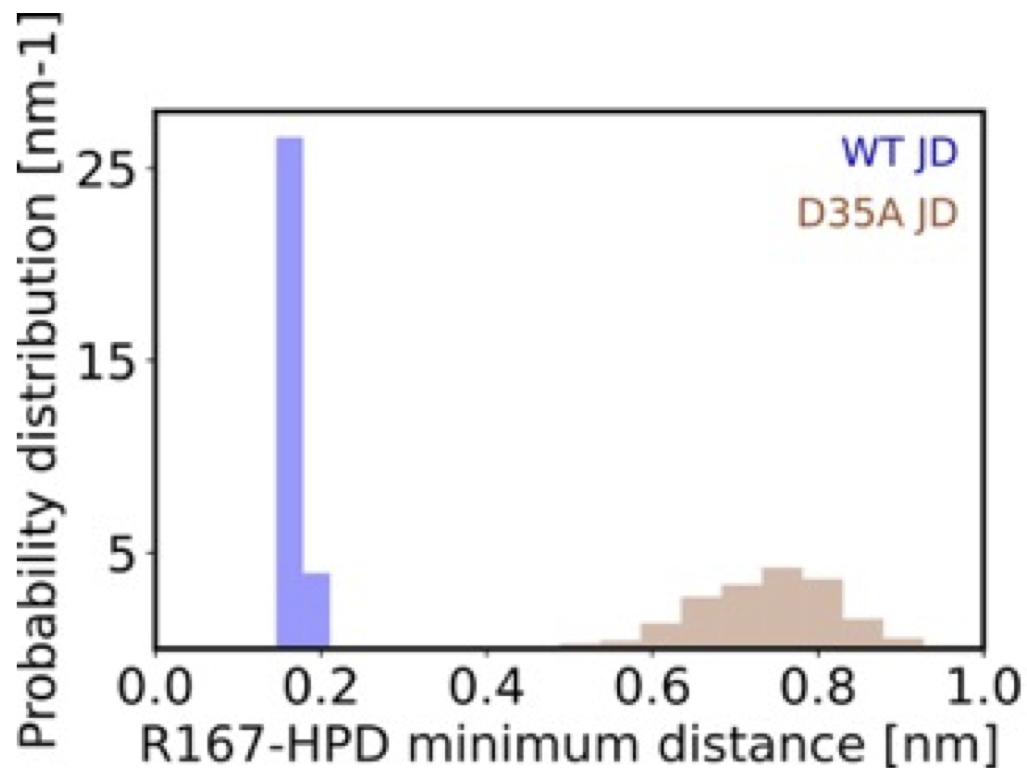

Supplement: S13 Fig — (TIF) [file pcbi.1014094.s012.tif]

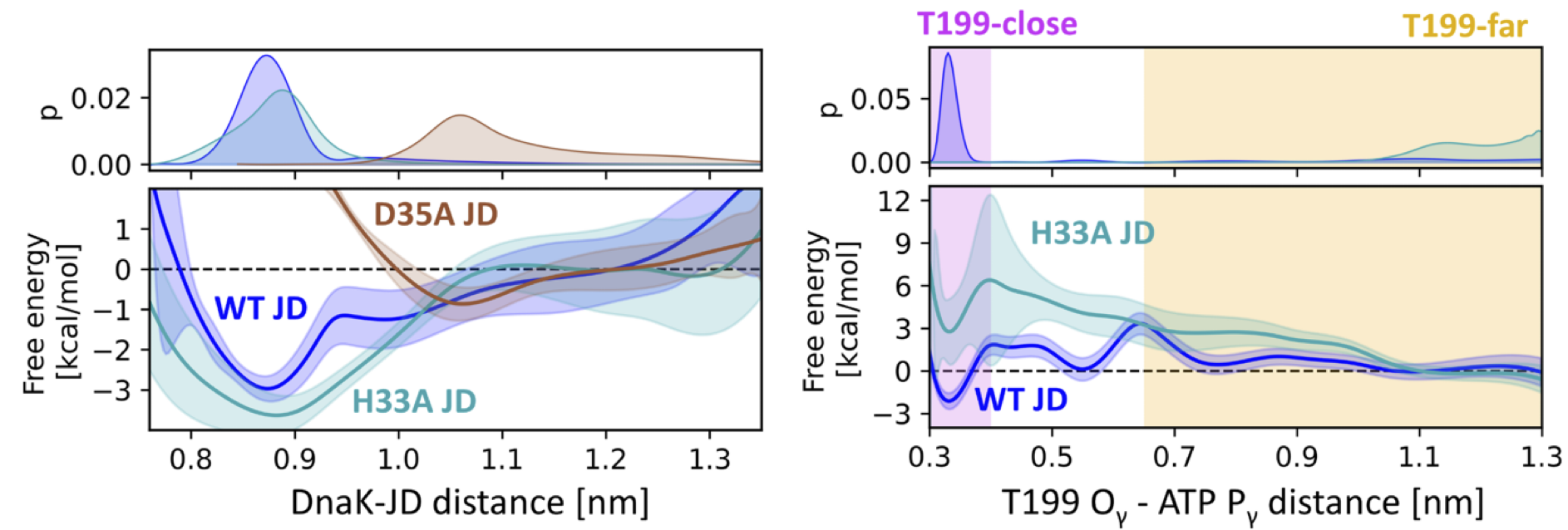

Supplement: S14 Fig — The profile for the H33A mutant was shown in cyan, profile for the D35A mutant was shown in brown, whereas profile for the wild-type JD binding was shown in blue. (Right) The probability distributions (top) and free energy profiles (bottom) for the T199-far to T199-close conformational change for the complex of H33A mutant (cyan) or the wild-type (blue) JD bound to Hsp70. (TIF) [file pcbi.1014094.s013.tif]

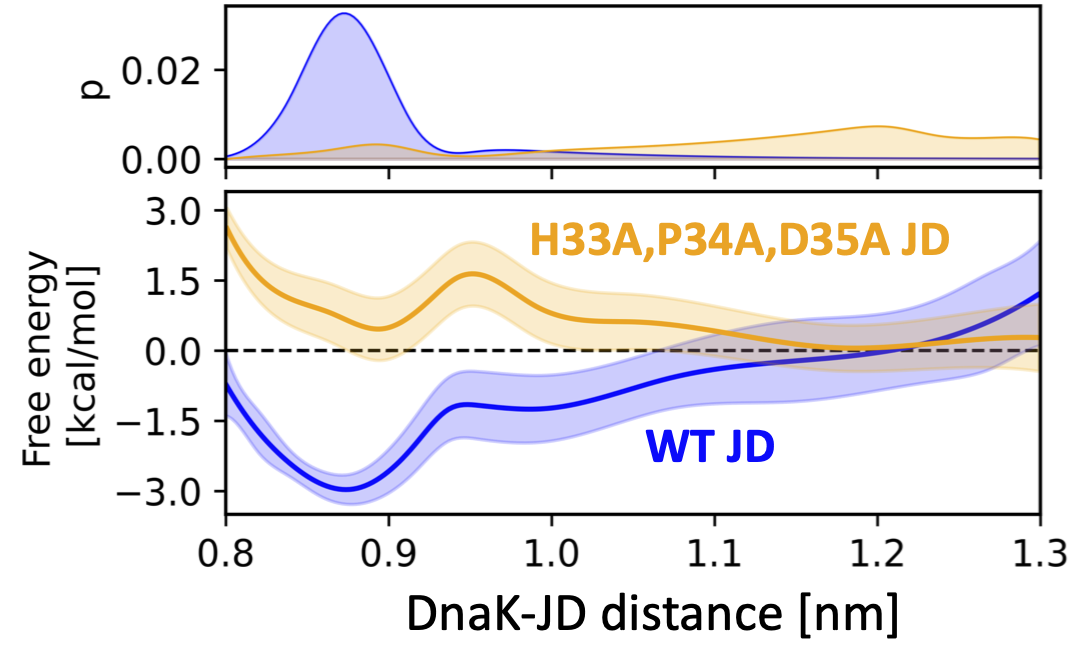

Supplement: S15 Fig — The profile for the H33A, P34A, D35A mutant was shown in orange, profile for the wild-type in blue. (TIF) [file pcbi.1014094.s014.tif]

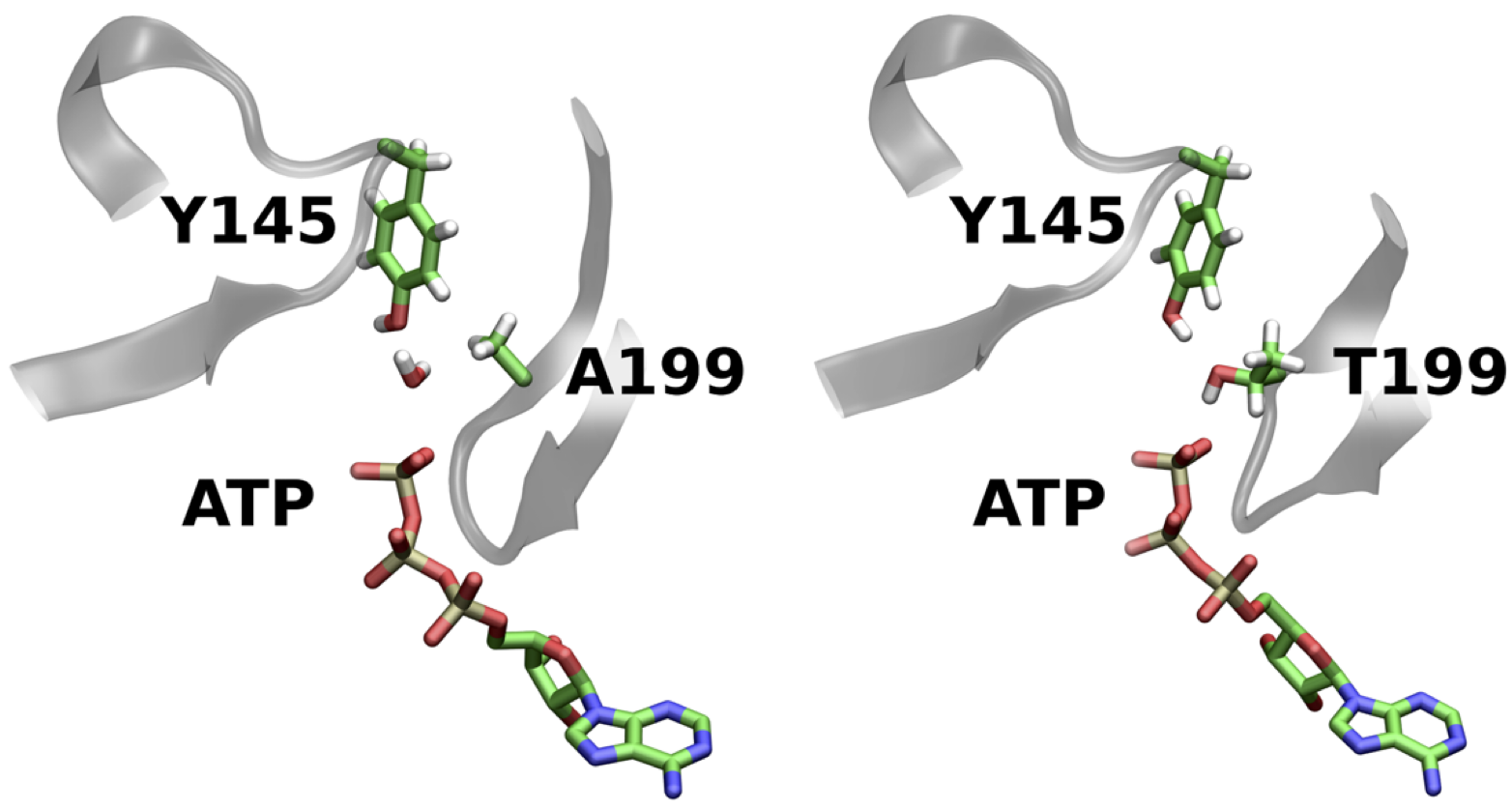

Supplement: S16 Fig — Profiles for the Hsp70 (blue) and Hsp70-JD (red) were added for comparison. (TIF) [file pcbi.1014094.s018.tif]

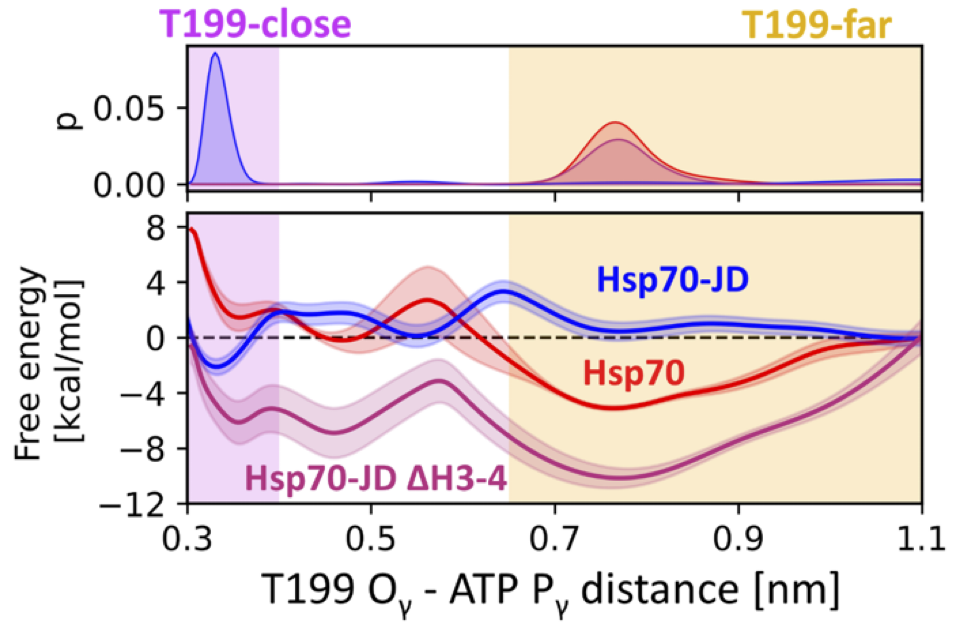

Supplement: S17 Fig — The non-biased profile for the formed β-sheet is shown in red, whereas the profile for the bias-induced disrupted β-sheet is shown in green. (Right) The probability distributions (top) and free energy profiles (bottom) for the T199-far to T199-close conformational change in the presence of the JD. The non-biased profile for the disrupted β-sheet is shown in blue, whereas the profile for the bias-induced β-sheet kept is shown in magenta. (TIF) [file pcbi.1014094.s015.tif]

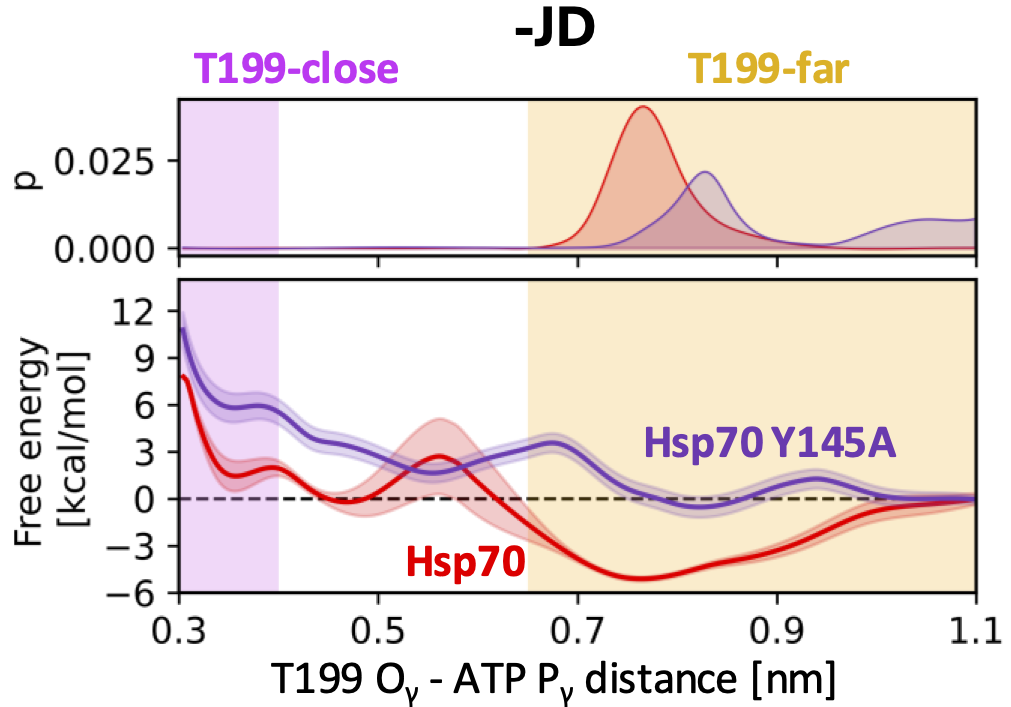

Supplement: S18 Fig — (TIF) [file pcbi.1014094.s019.tif]

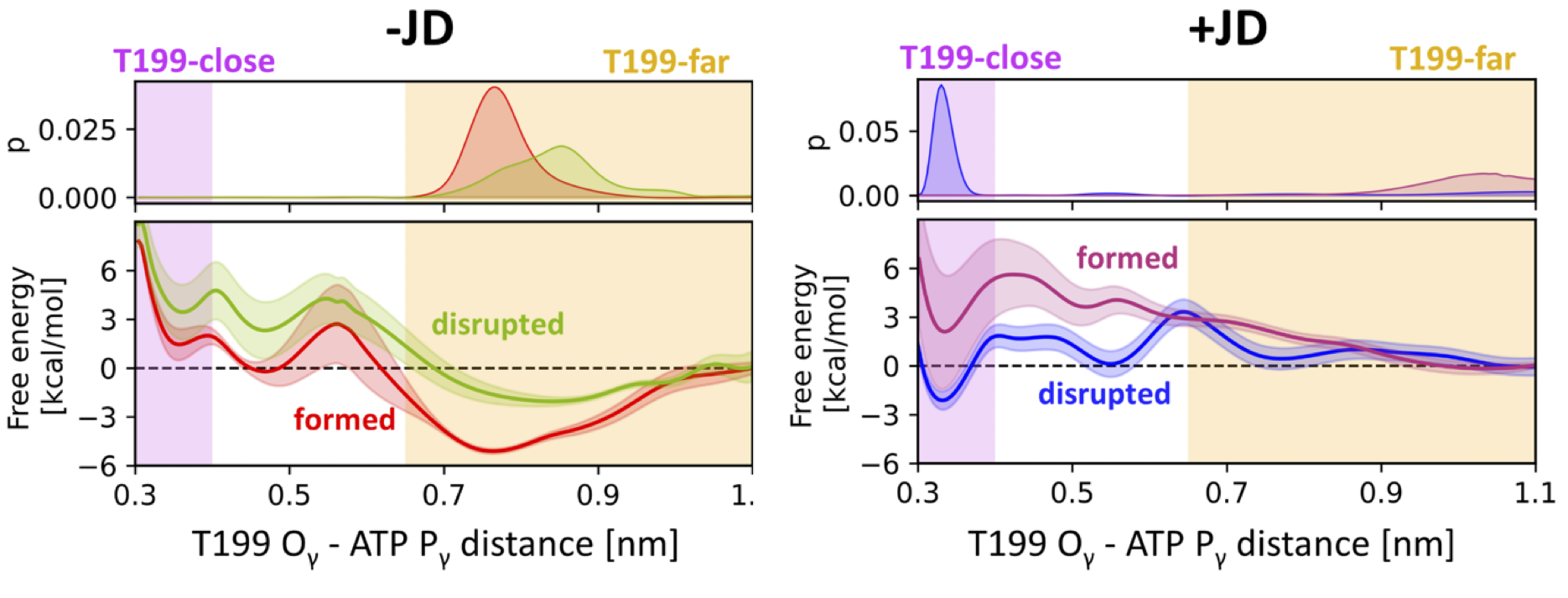

Supplement: S19 Fig — (A) Structural representation of the movement of T199 towards the ATP enabled by beta-sheet disruption. The ATP and T199 are shown in licoricey representation, whereas beta-sheet is shown in cartoon representation. (B) Probability distribution of the antiparallel beta-sheet content of the 198–206 and 219–227 residues of Hsp70 in the Hsp70 alone (red) and the Hsp70-JD complex (blue). (TIF) [file pcbi.1014094.s016.tif]

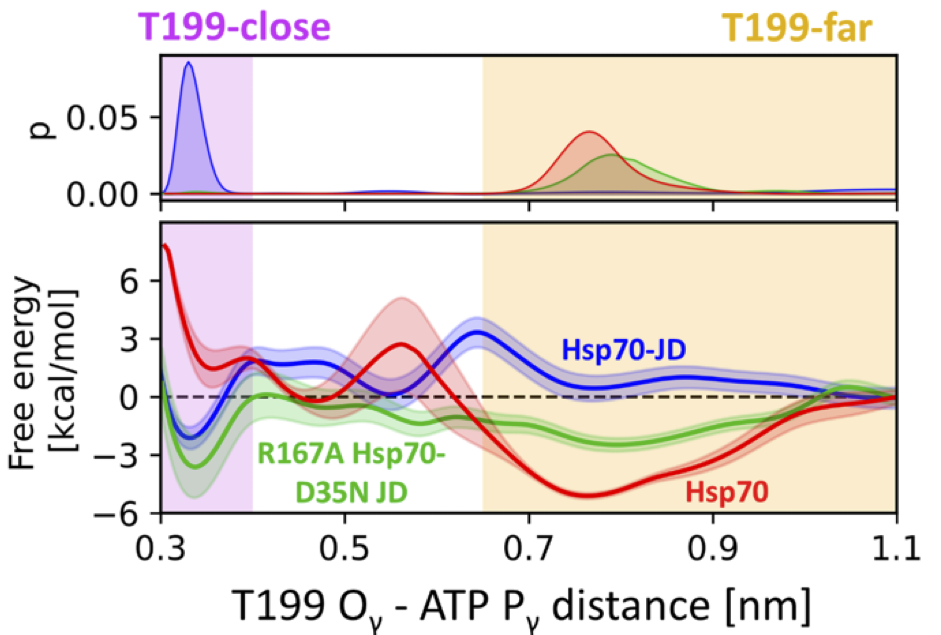

Supplement: S20 Fig — (TIF) [file pcbi.1014094.s020.tif]

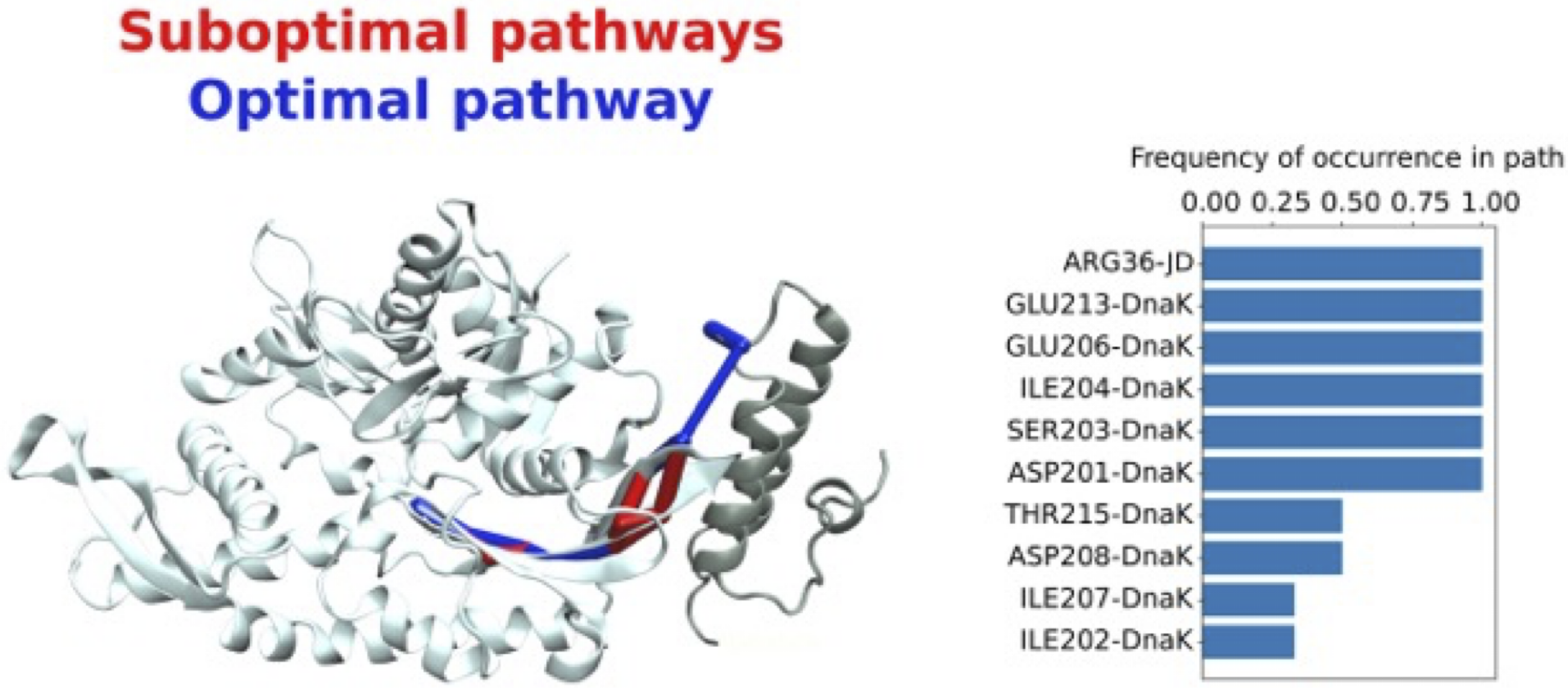

Supplement: S21 Fig — (TIF) [file pcbi.1014094.s021.tif]

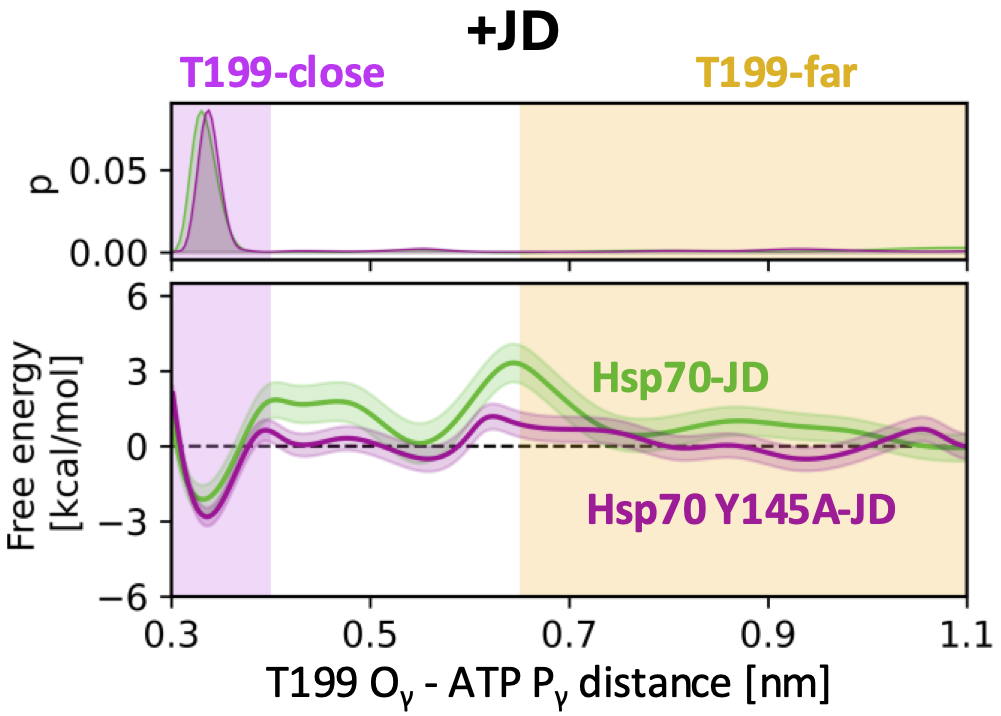

Supplement: S22 Fig — (TIF) [file pcbi.1014094.s022.tif]

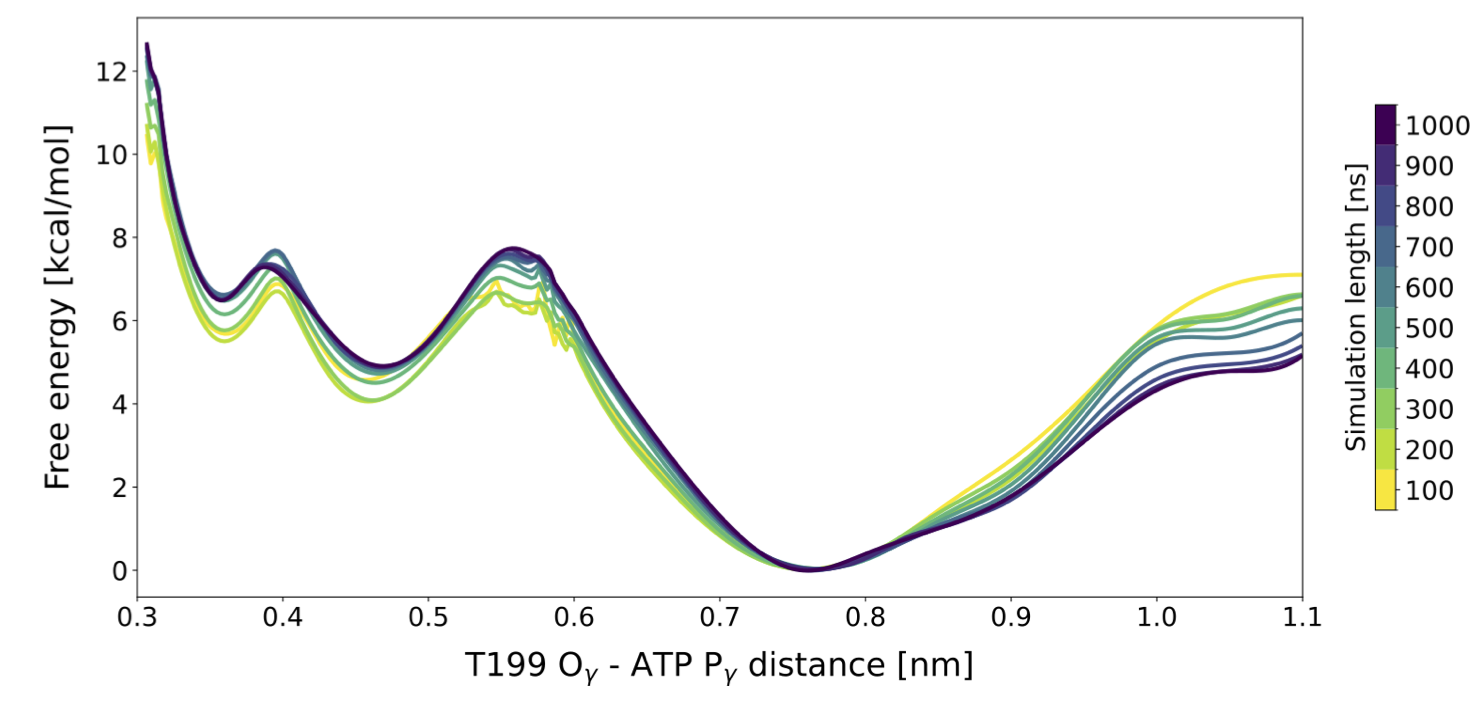

Supplement: S23 Fig — Profiles for wildtype complex (blue) and Hsp70 alone (red) added for comparison. (TIF) [file pcbi.1014094.s023.tif]

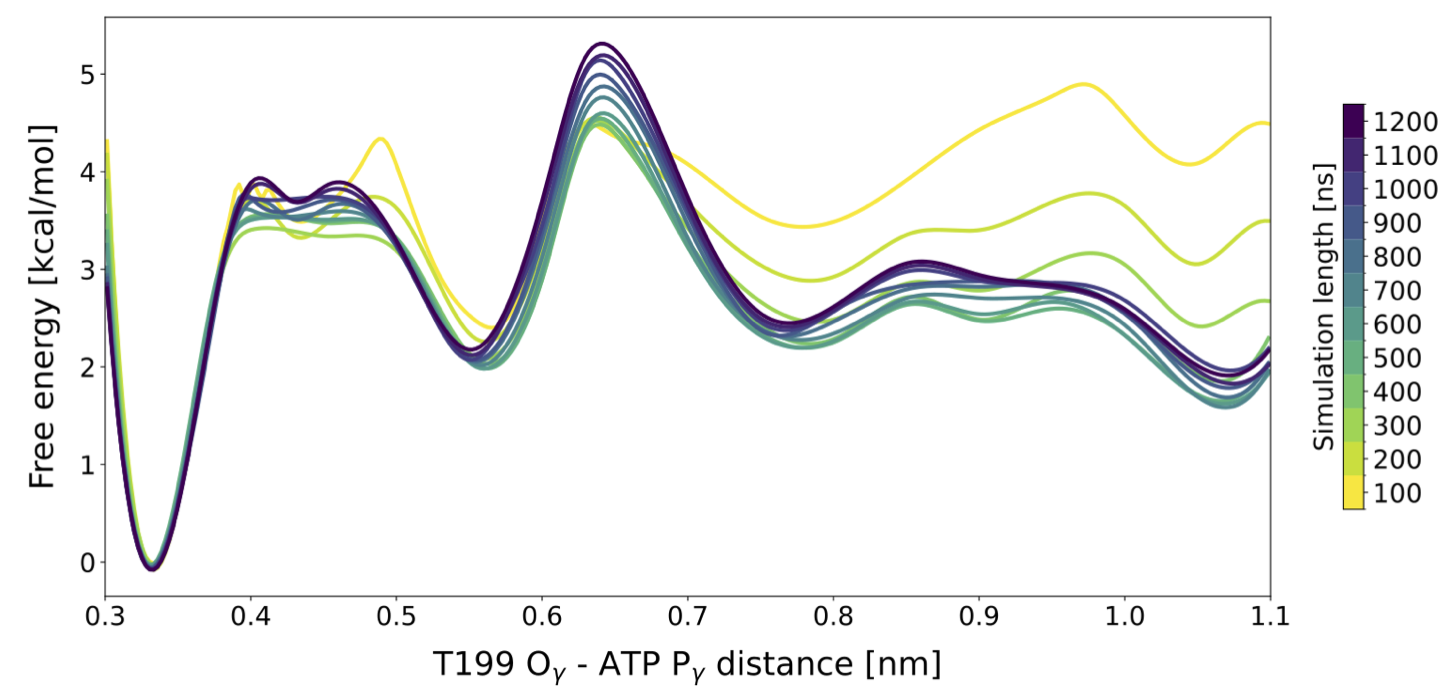

Supplement: S24 Fig — The pathways between HPD motif of JD and the catalytic pocket of Hsp70 were calculated from the Umbrella Sampling trajectories corresponding to the T199-close state. The optimal pathway was indicated in blue, whereas suboptimal pathways were depicted in red. (TIF) [file pcbi.1014094.s024.tif]

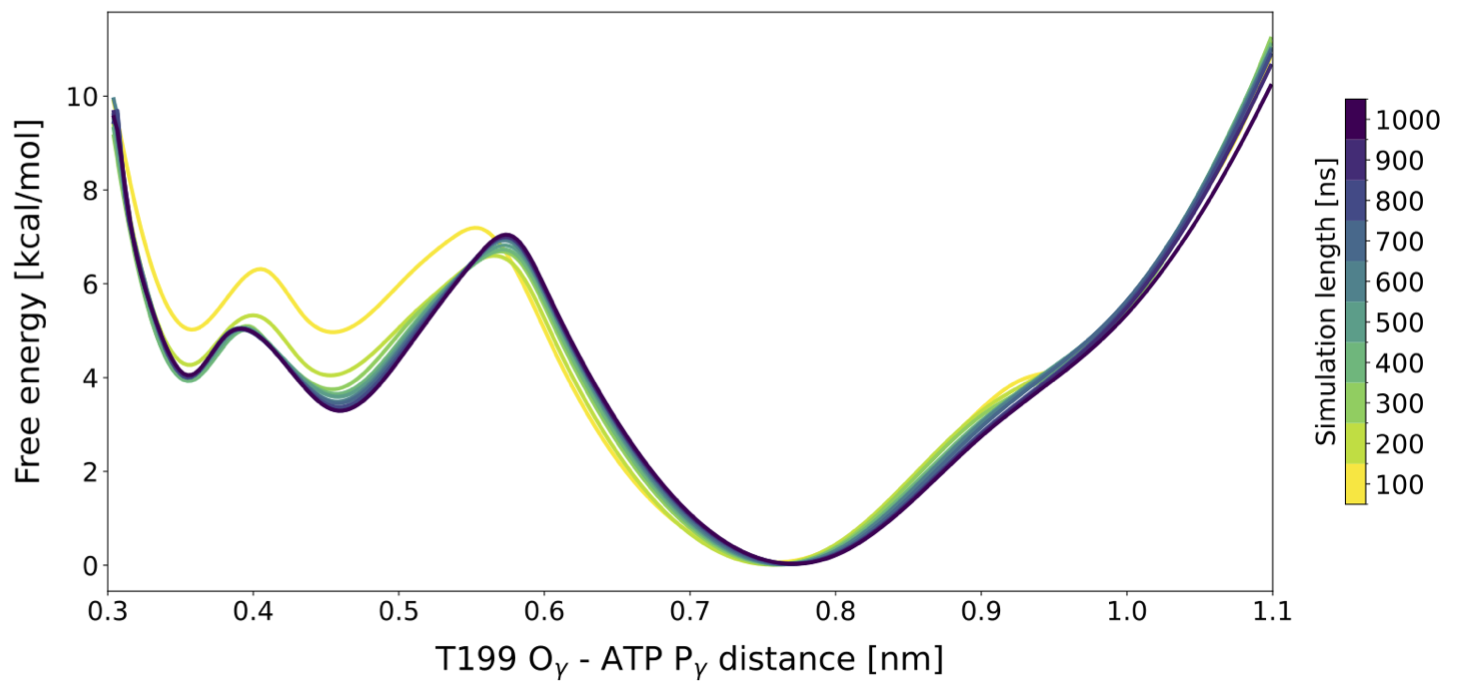

Supplement: S25 Fig — (TIF) [file pcbi.1014094.s026.tif]

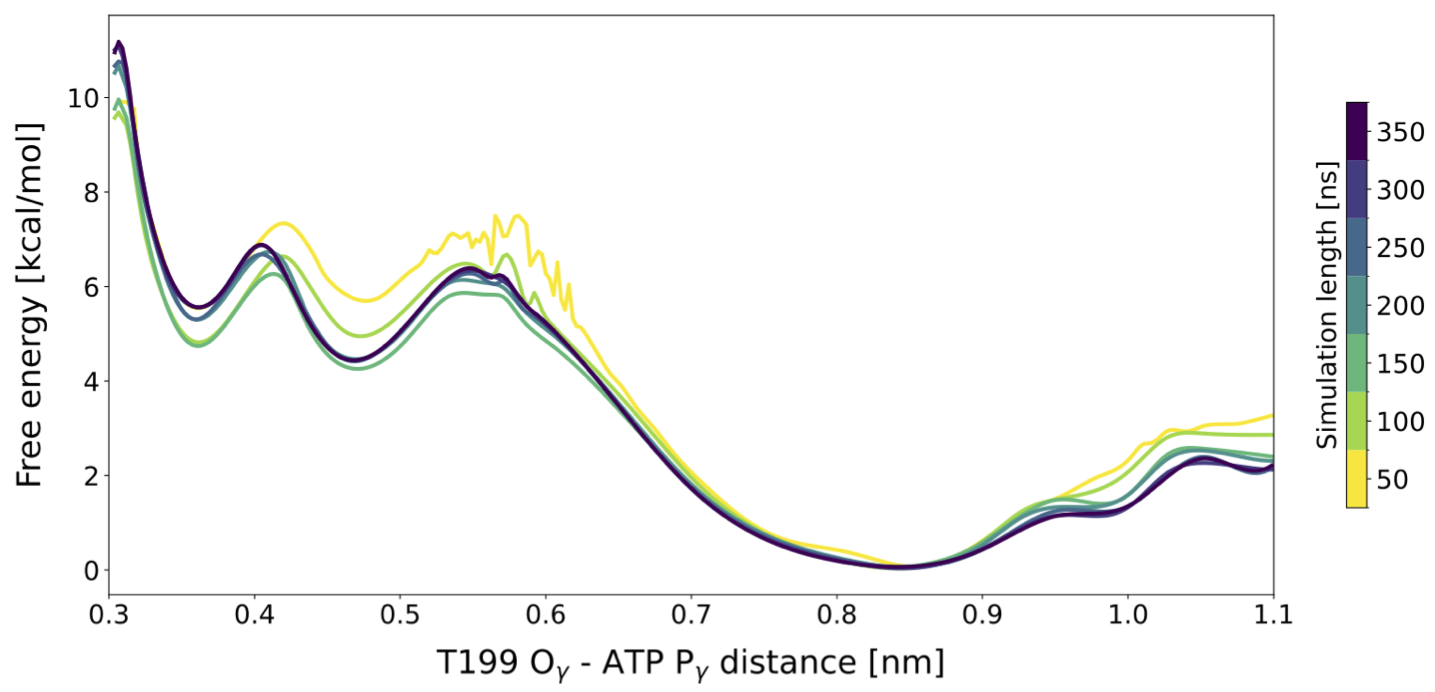

Supplement: S26 Fig — (TIF) [file pcbi.1014094.s027.tif]

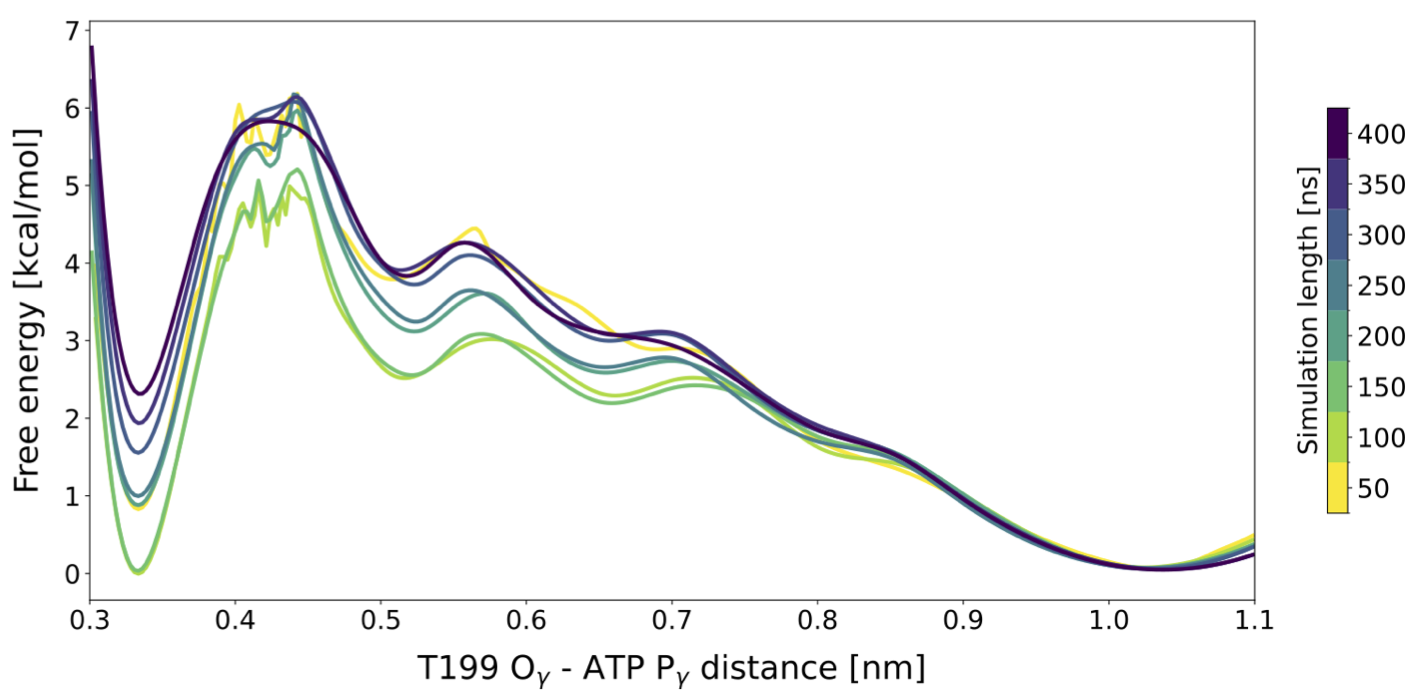

Supplement: S27 Fig — (TIF) [file pcbi.1014094.s028.tif]

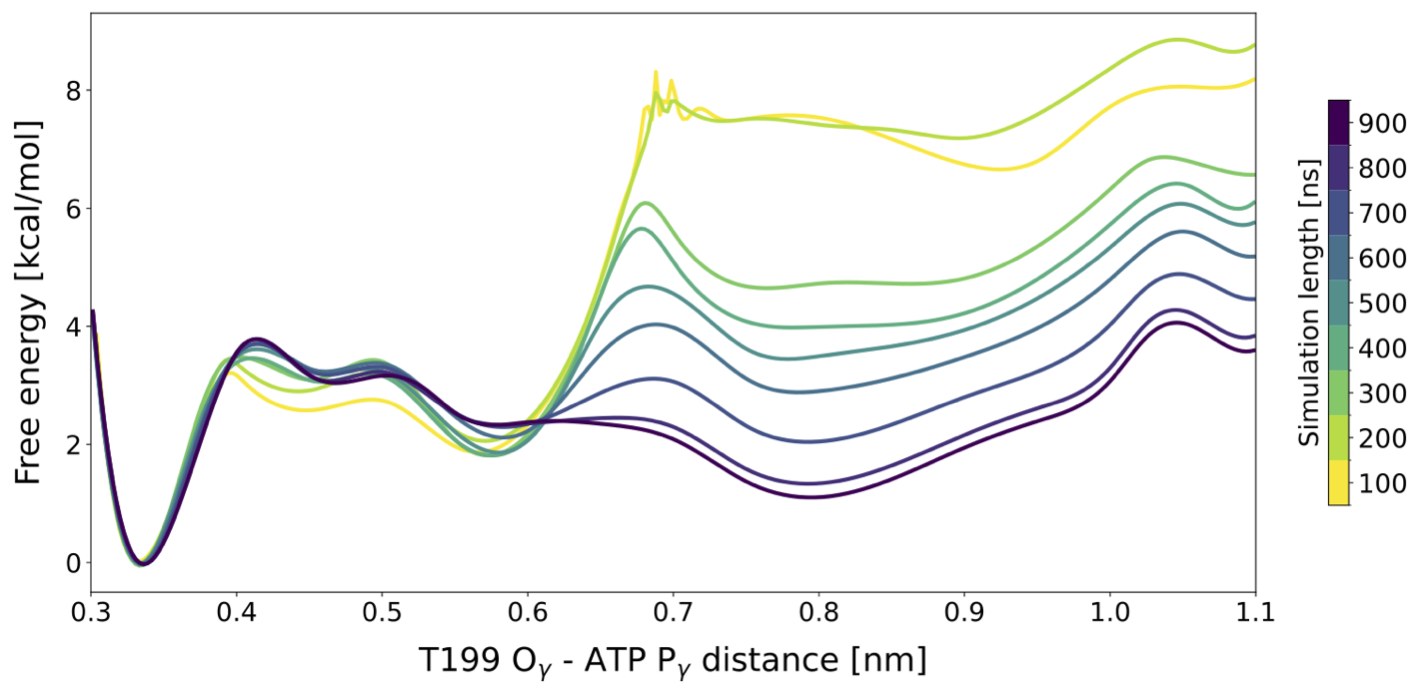

Supplement: S28 Fig — (TIF) [file pcbi.1014094.s029.tif]

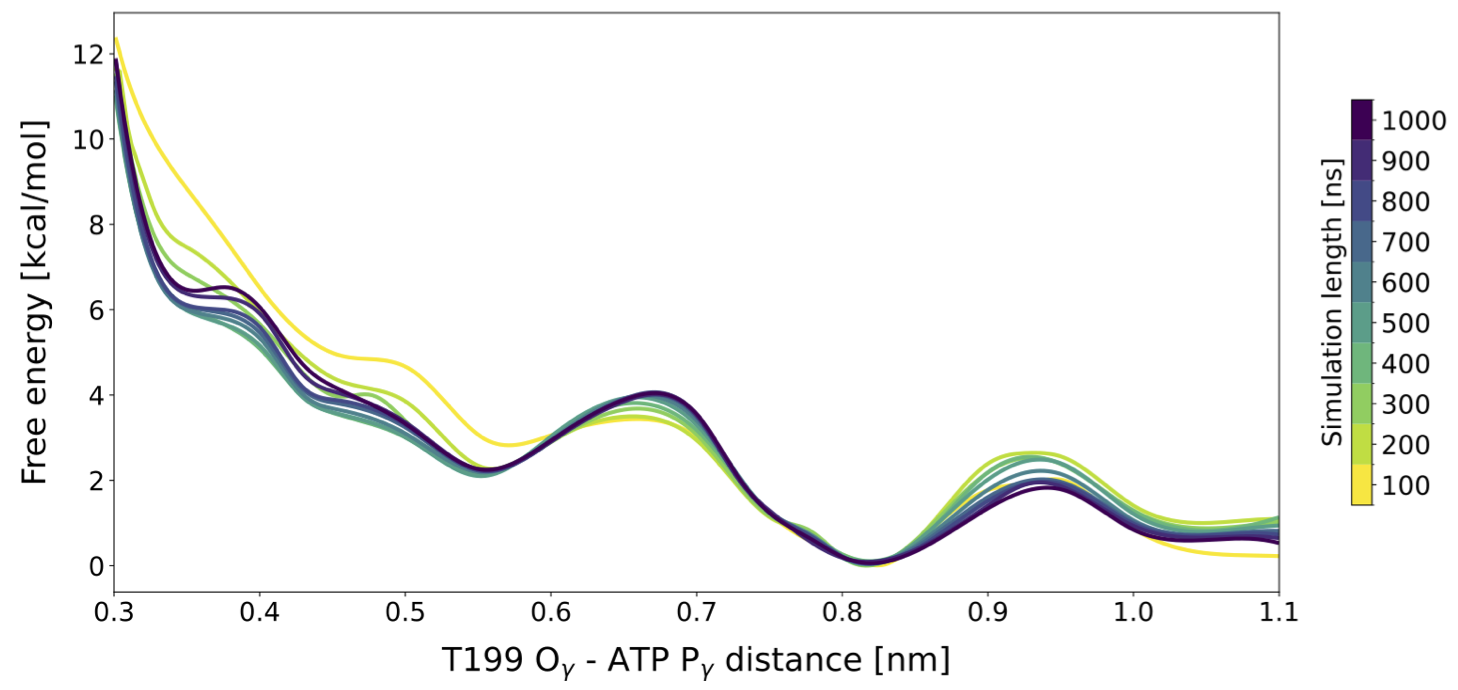

Supplement: S29 Fig — (TIF) [file pcbi.1014094.s030.tif]

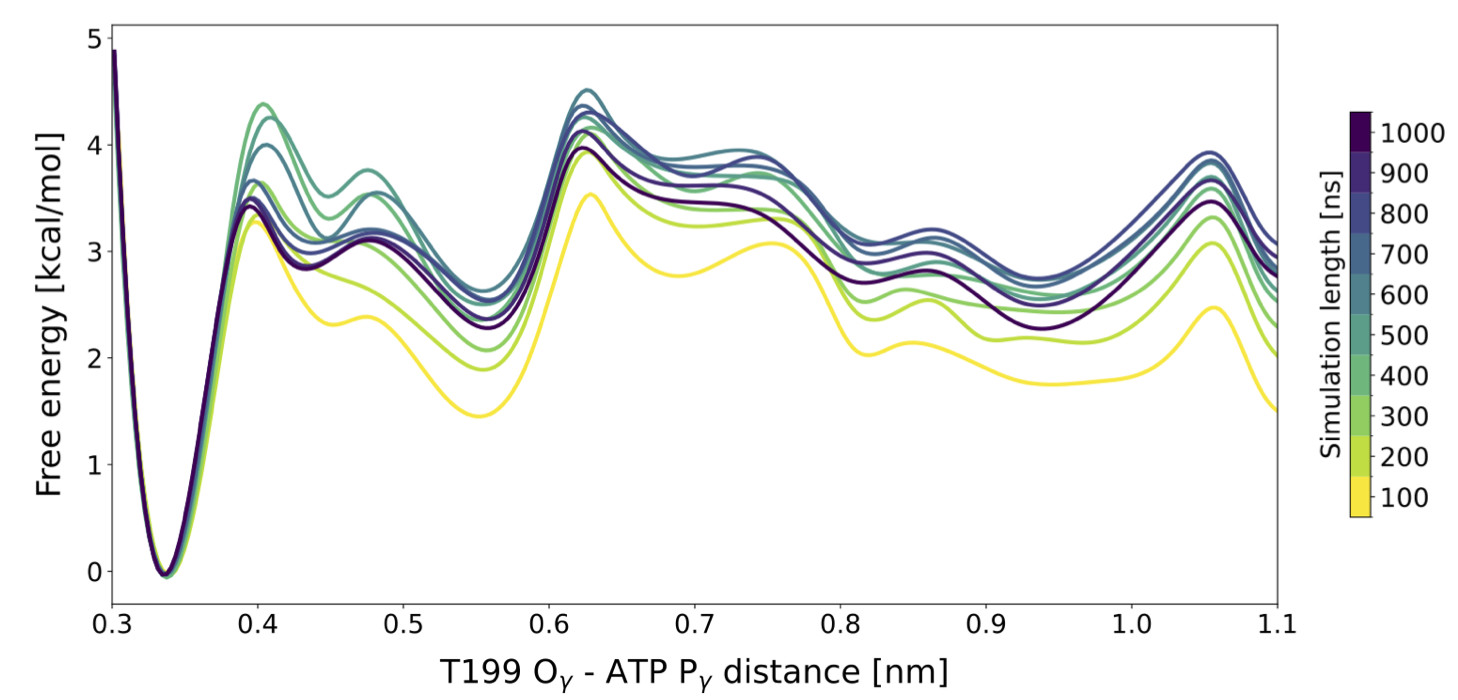

Supplement: S30 Fig — (TIF) [file pcbi.1014094.s031.tif]

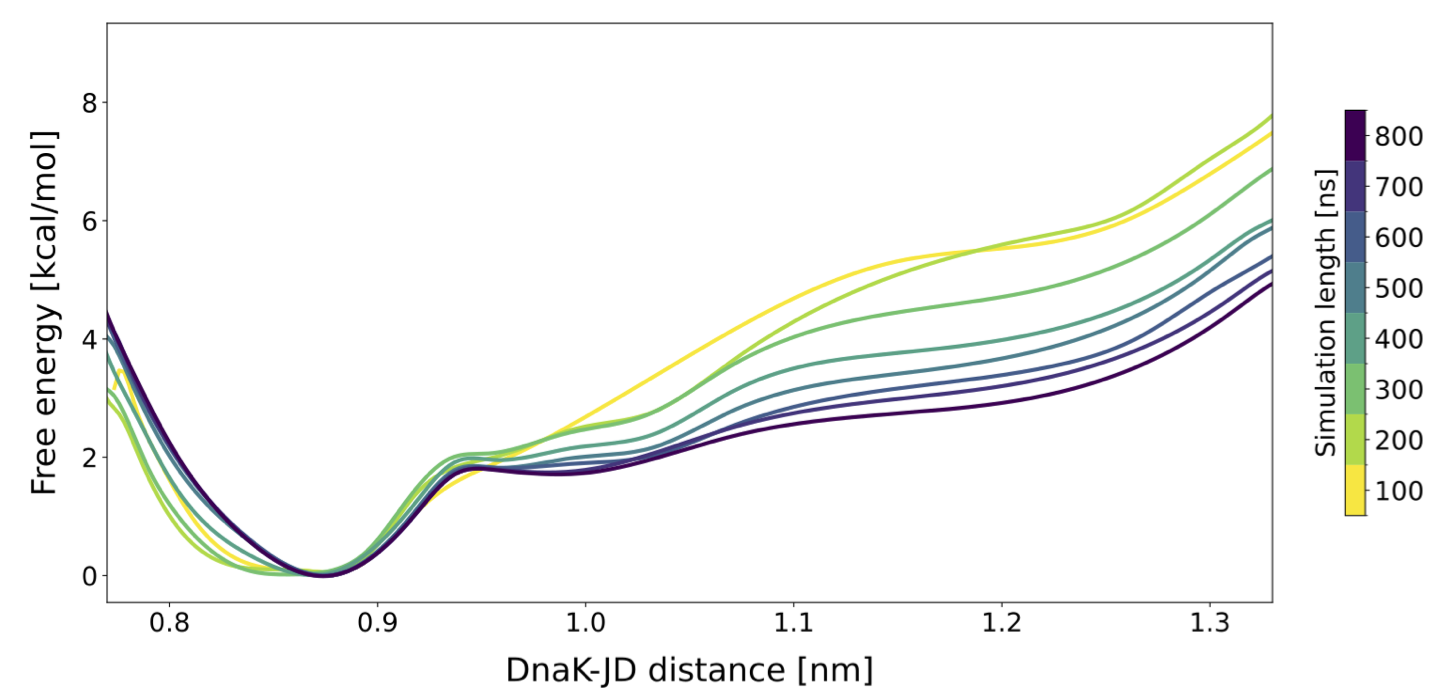

Supplement: S31 Fig — (TIF) [file pcbi.1014094.s032.tif]

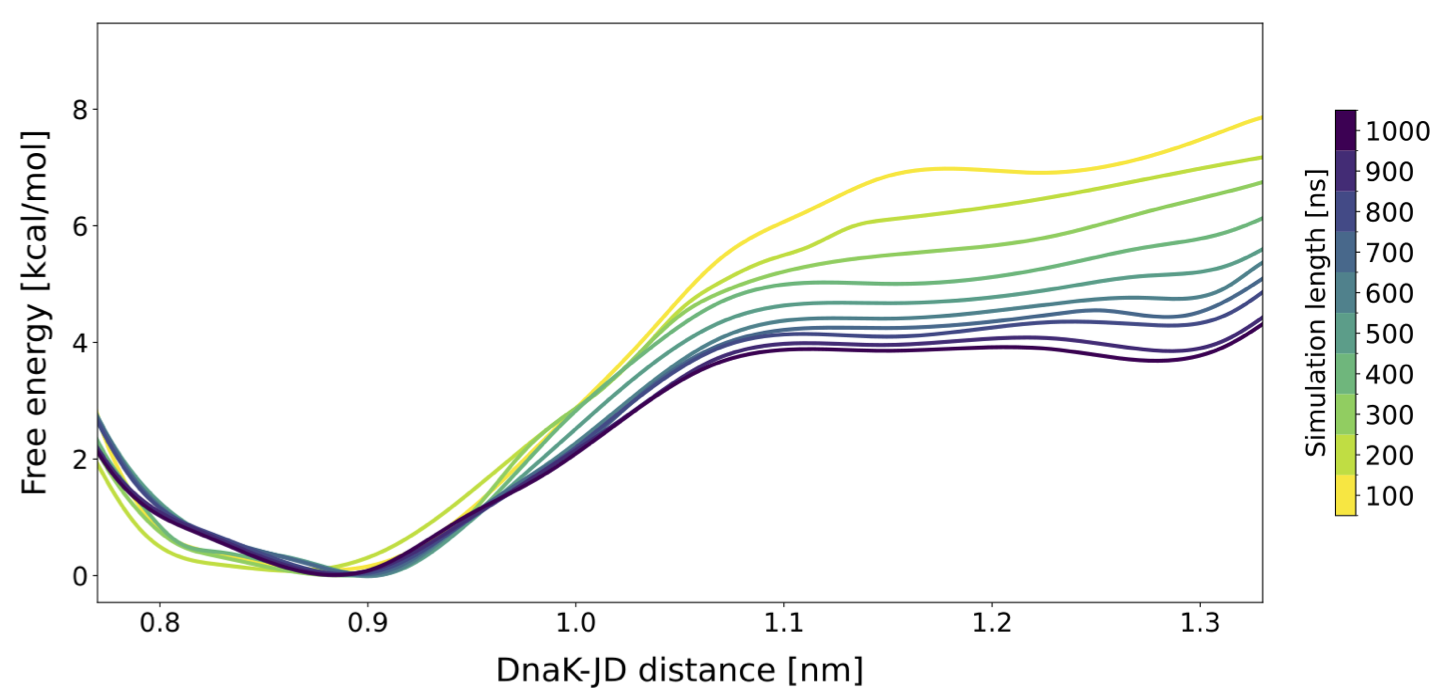

Supplement: S32 Fig — (TIF) [file pcbi.1014094.s033.tif]

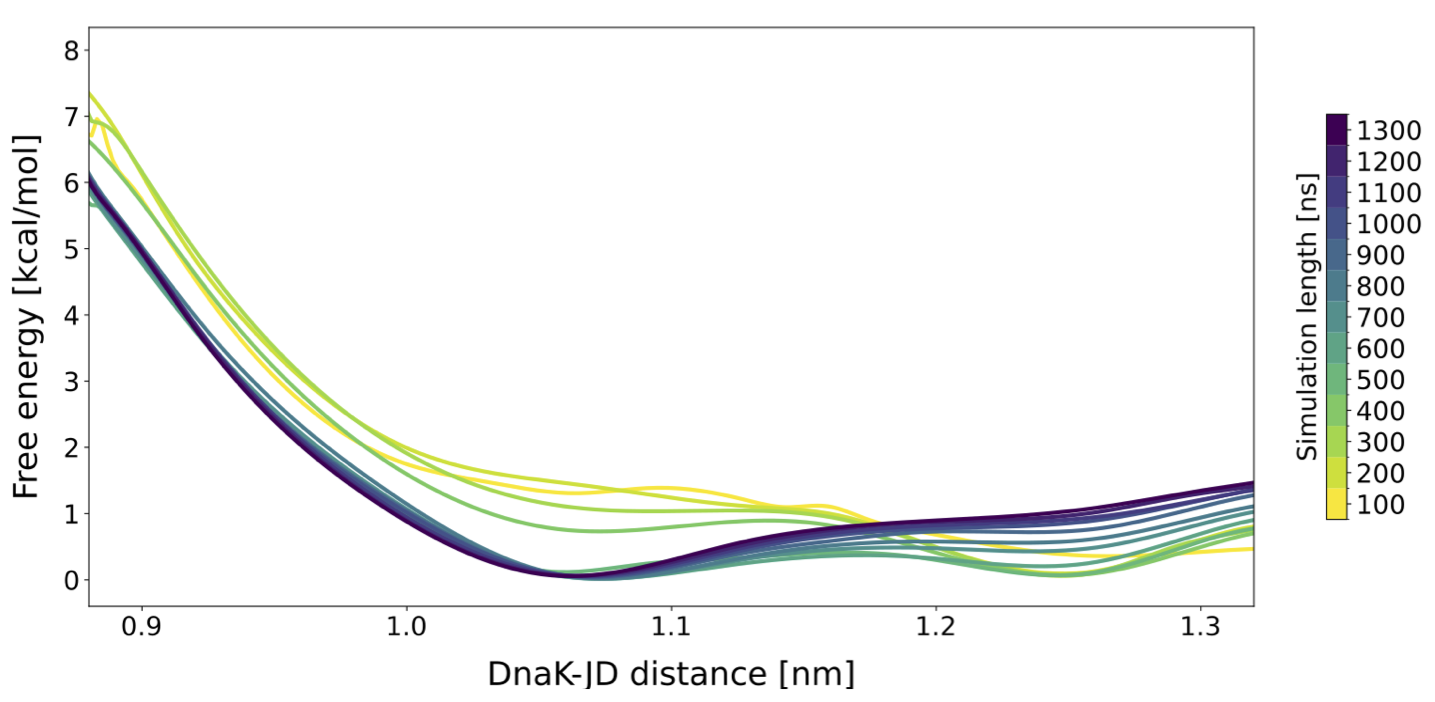

Supplement: S33 Fig — (TIF) [file pcbi.1014094.s034.tif]

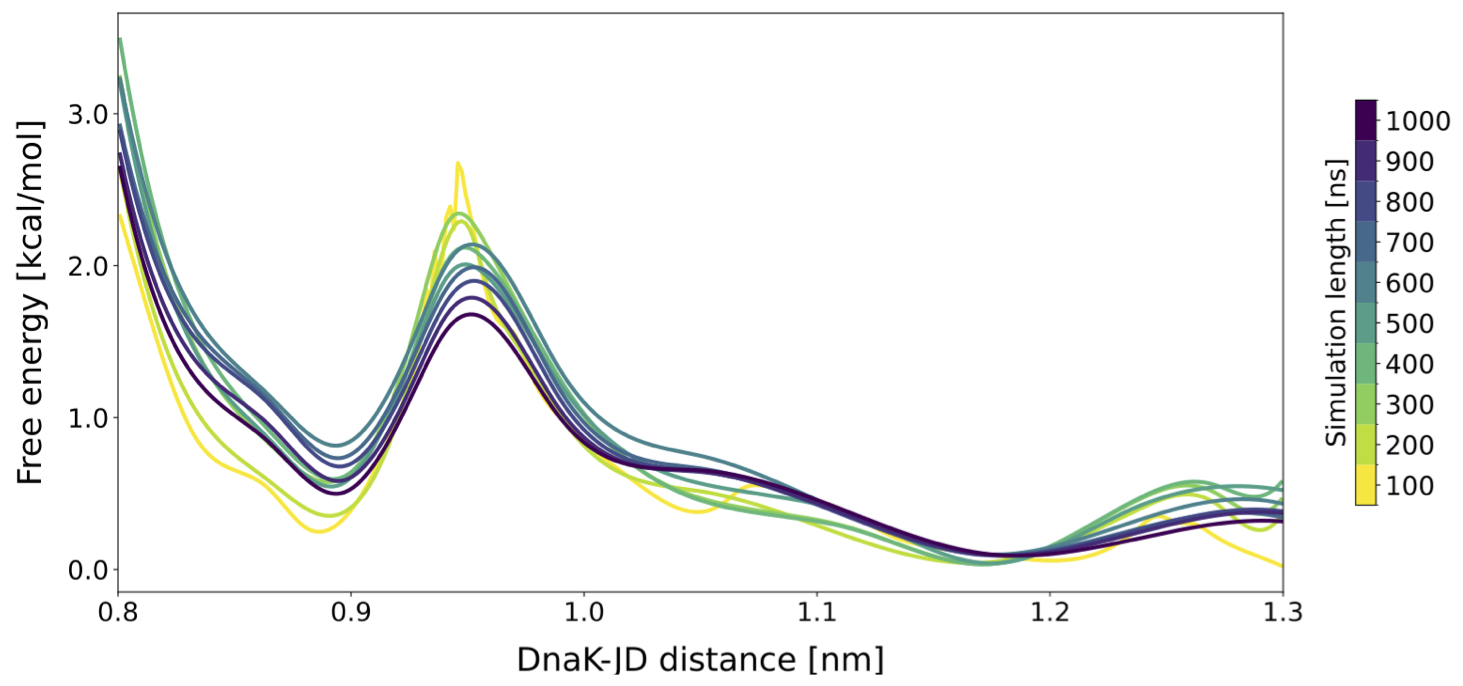

Supplement: S34 Fig — (TIF) [file pcbi.1014094.s035.tif]

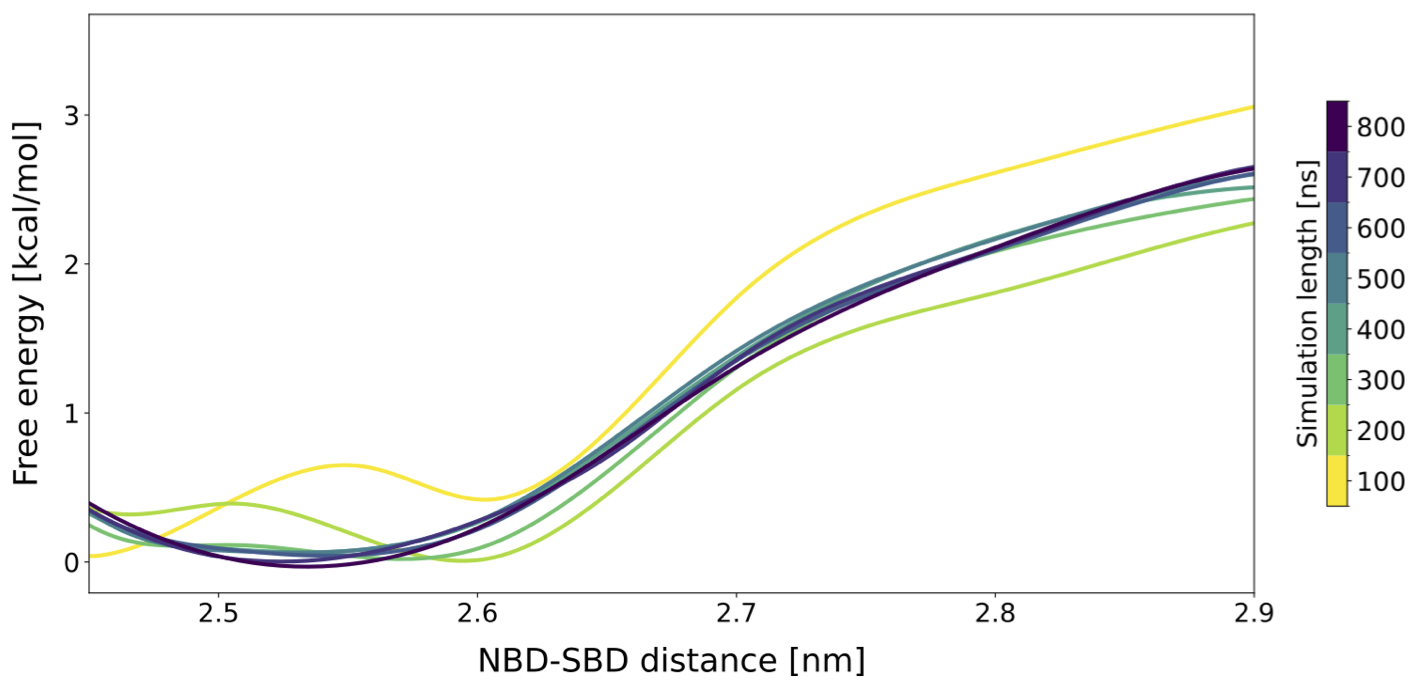

Supplement: S35 Fig — (TIF) [file pcbi.1014094.s036.tif]

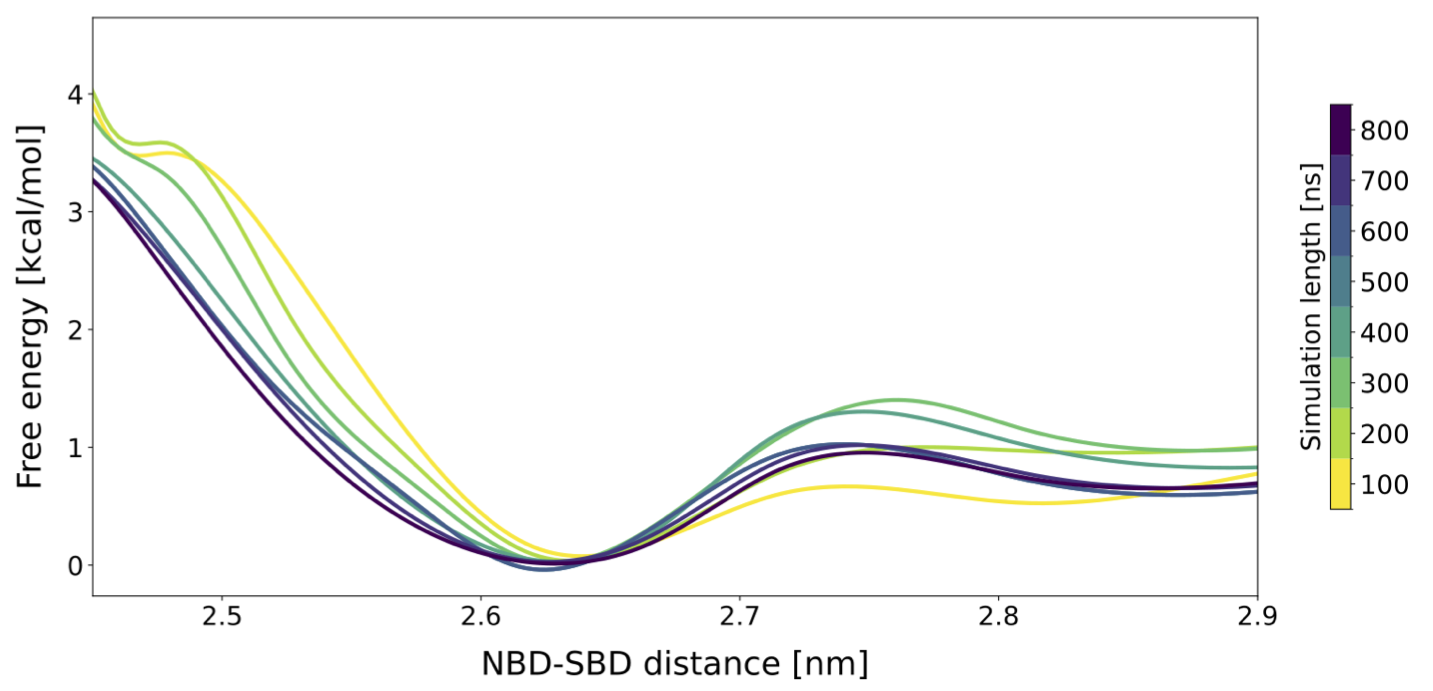

Supplement: S36 Fig — (TIF) [file pcbi.1014094.s037.tif]

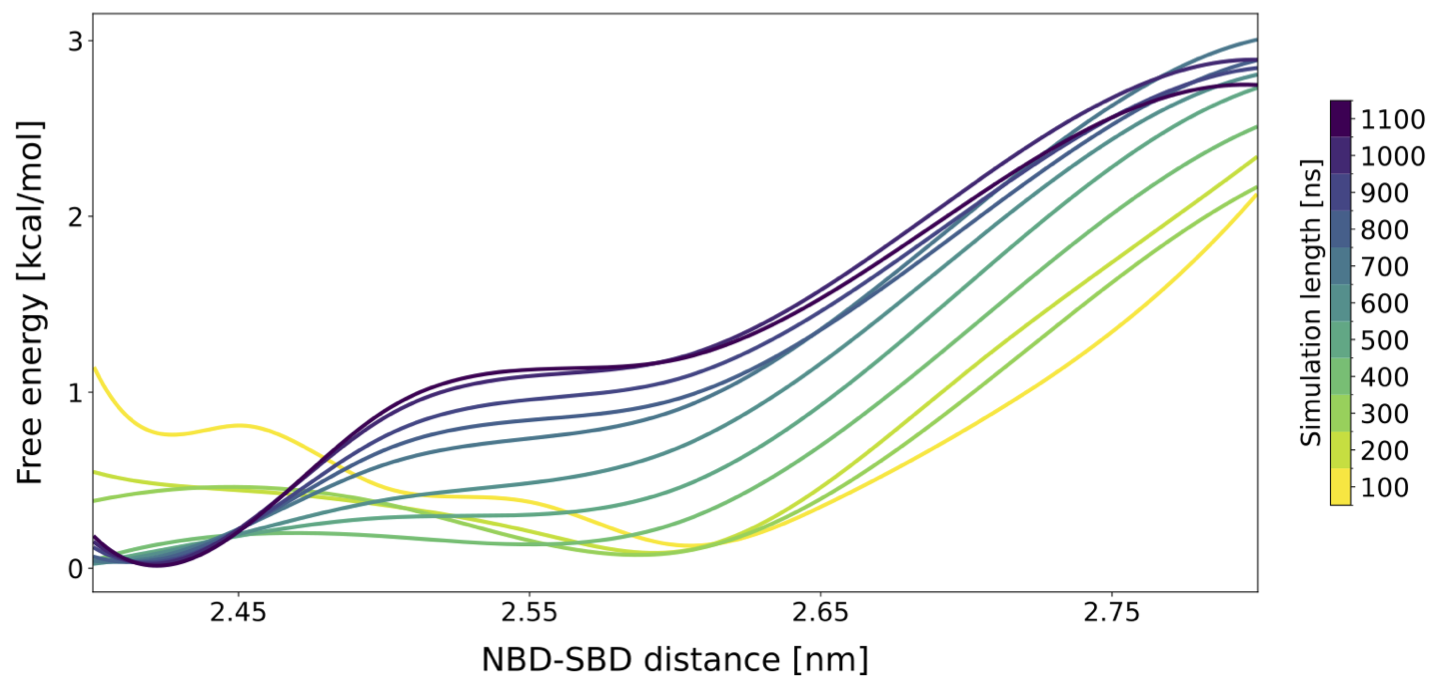

Supplement: S37 Fig — (TIF) [file pcbi.1014094.s038.tif]

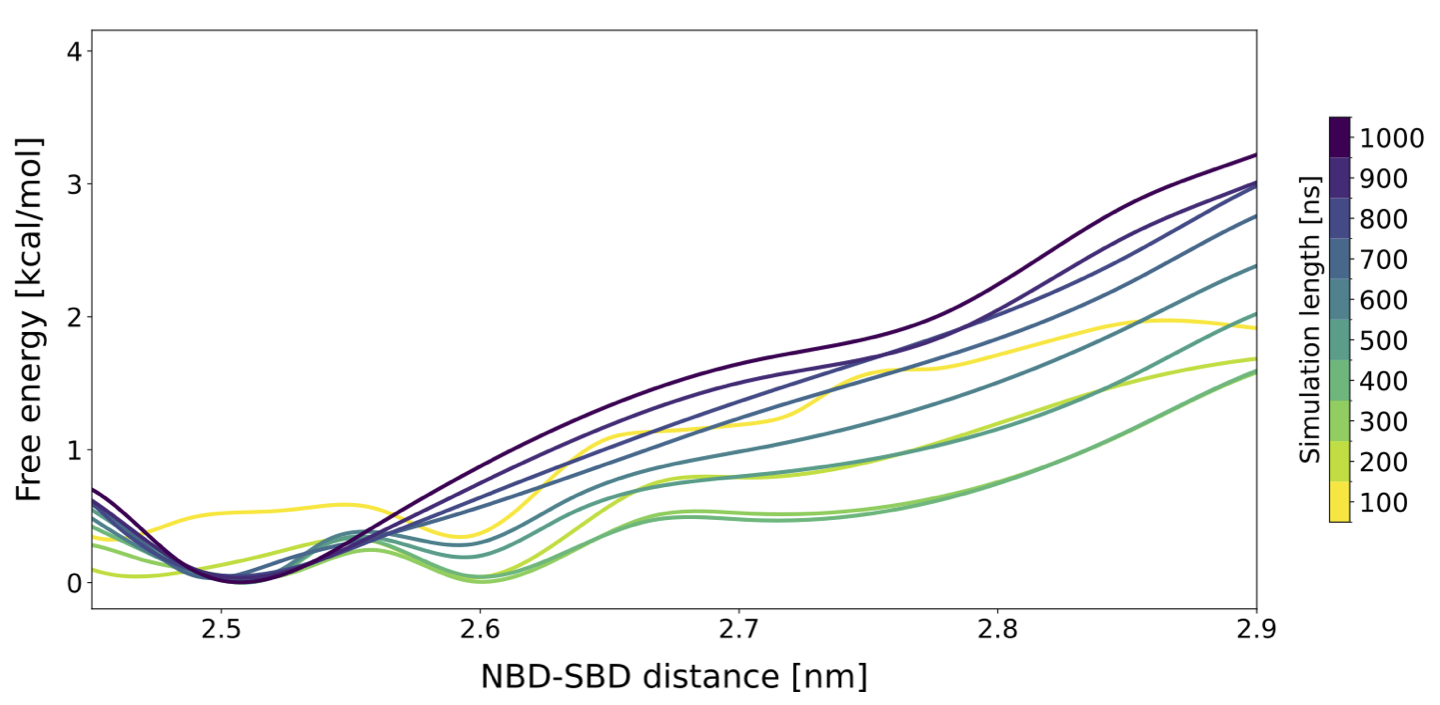

Supplement: S38 Fig — (TIF) [file pcbi.1014094.s039.tif]

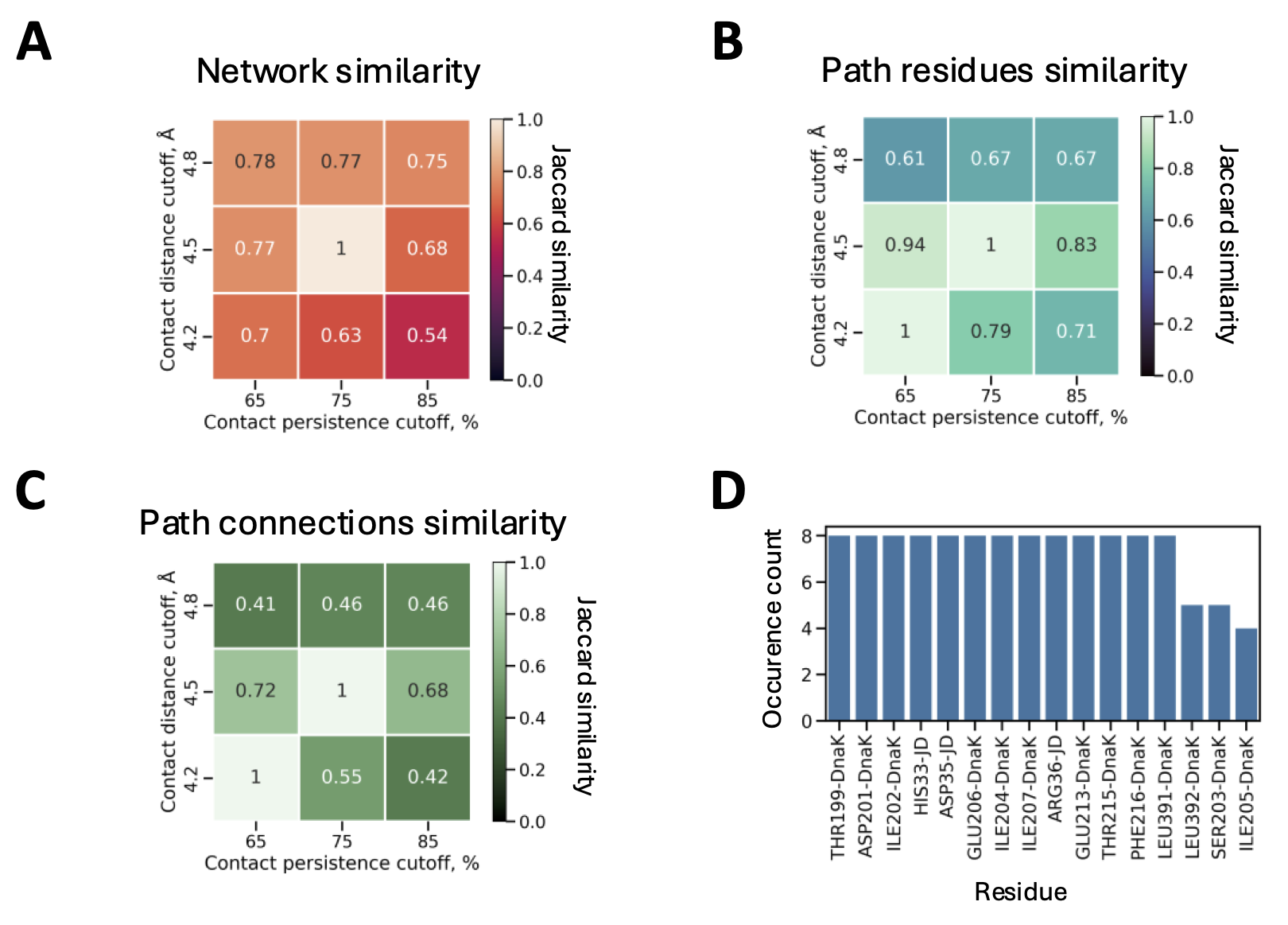

Supplement: S39 Fig — (TIF) [file pcbi.1014094.s040.tif]

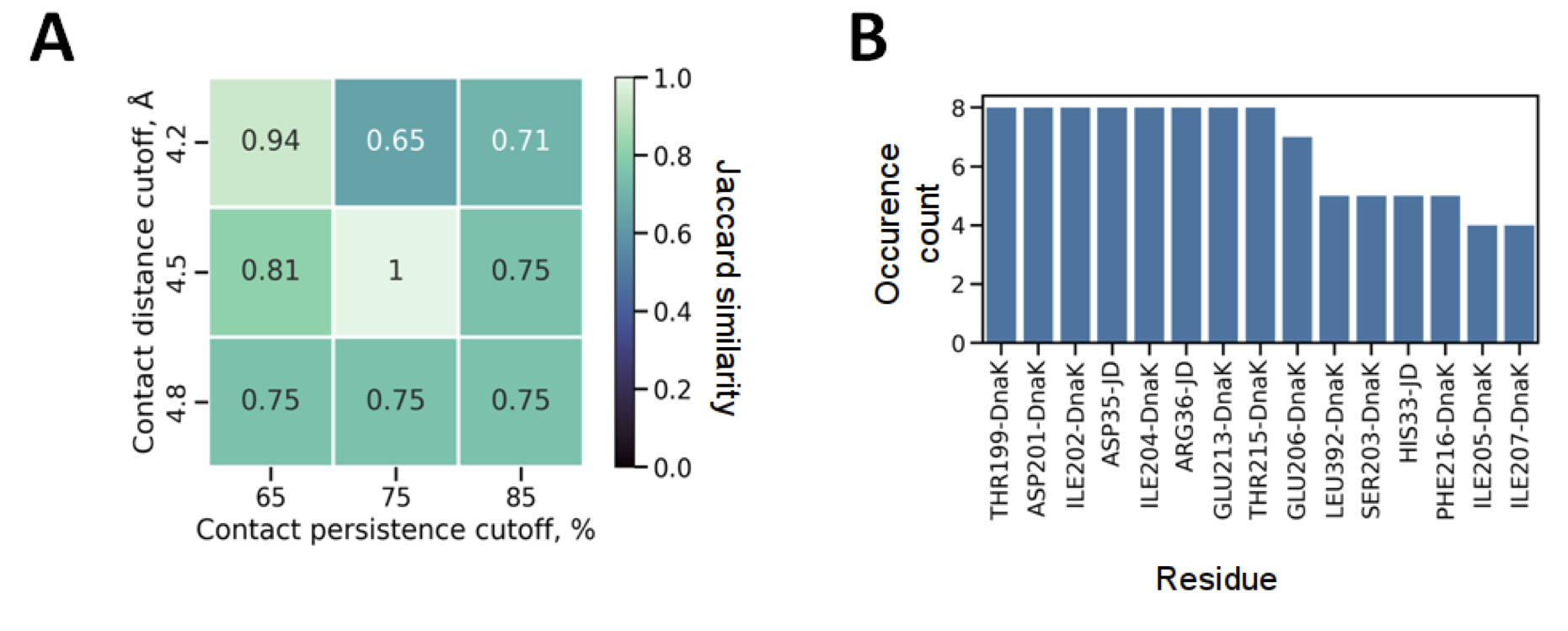

Supplement: S40 Fig — (TIF) [file pcbi.1014094.s041.tif]

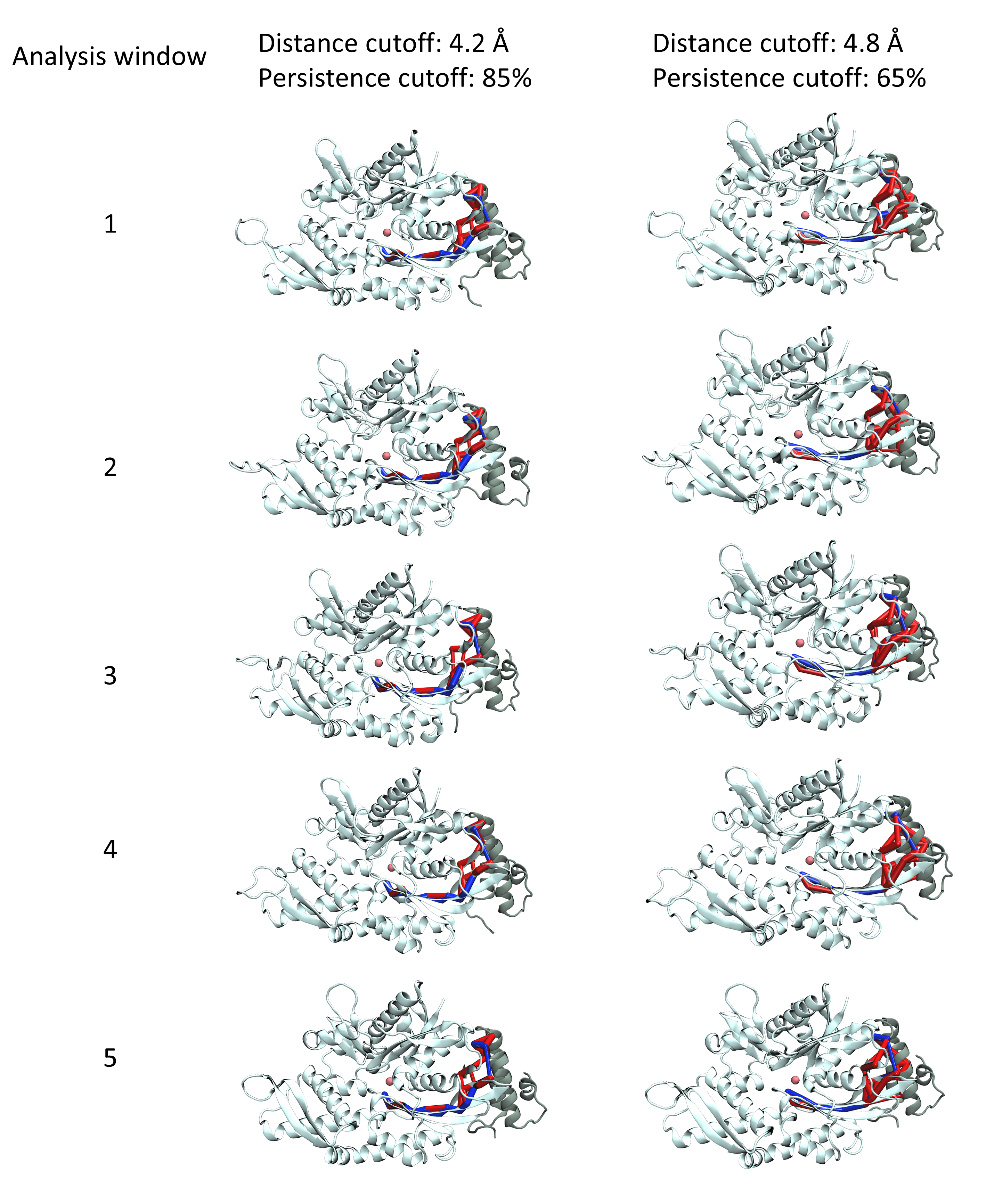

Supplement: S41 Fig — (TIF) [file pcbi.1014094.s042.tif]

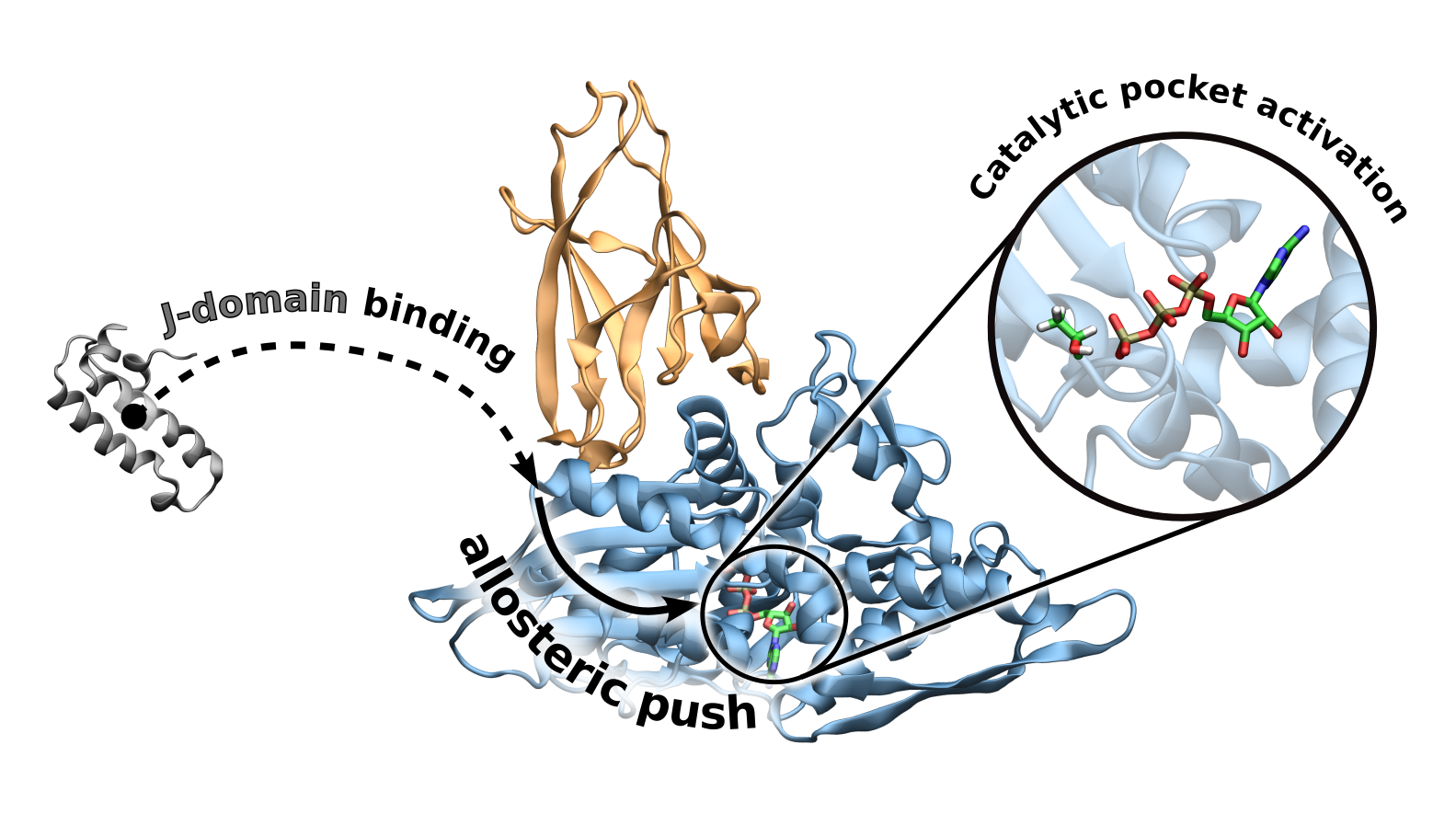

Supplement: S45 Fig — (TIFF) [file pcbi.1014094.s046.tiff]
